# Optimization of *Spirulina*‐Enriched Vegan Cake Formulation Using Response Surface Methodology

**DOI:** 10.1002/fsn3.70116

**Published:** 2025-03-24

**Authors:** Eda Nurko, Emine Nakilcioğlu

**Affiliations:** ^1^ Ege University, Engineering Faculty Food Engineering Department Izmir Türkiye

**Keywords:** optimization, response surface methodology, *spirulina*, vegan cake

## Abstract

Vegan lifestyle is gaining momentum across the globe due to its environmental effects and health benefits. In parallel with the increasing diet trends, the demand for vegan bakery products is increasing. Since vegan bakery products generally have low protein and bioactive compound content, they have little nutritional contribution to the daily diet of vegan individuals. In light of this, a study was conducted to develop a vegan cake formulation enriched with *Spirulina* to improve the physical, nutritional, and sensory properties of the product. Response surface methodology (RSM) was used to determine the optimum formulation for the production of *Spirulina*‐enriched vegan cakes. The effects of *Spirulina* content (5–15 g), sugar content (90–110 g), flour content (90–110 g), and fat content (25–40 g) on some chemical, physical, and sensory properties of vegan cakes were investigated. It was found that the amount of *Spirulina*, the amount of sugar, the amount of flour, and the amount of fat could significantly affect the responses (*p* < 0.05). The optimum values for the independent variables were 11.965 g *Spirulina*, 106.206 g sugar, 110 g flour, and 25 g oil. The optimum formulation confirmed the fit of the regression models. In the optimum formulation of vegan cake enriched with *Spirulina*, baking loss was found to be 11.22%, hardness to be 43.96 N, Lcrumb* value to be 37.54, L_crust_* value to be 41.94, protein content to be 4.2%, total phenolic content to be 186.475 mg GAE/100 g DW, ABTS antioxidant activity to be 15.5679 μmol TE/100 g DW, and overall acceptability value to be 8.2. It is thought that vegan cake enriched with *Spirulina* can create a new trend for vegan individuals. Additionally, the developed product stands out as a nutritious alternative to vegan cakes on the bakery market.

## Graphical abstract

This study aimed to develop a *Spirulina*‐enriched vegan cake formulation using RSM. The optimum formulation of vegan cake was found to be 11.965 g *Spirulina*, 106.206 g sugar, 110 g flour, and 25 g oil. In the vegan cake, baking loss was determined as 11.22%, hardness as 43.96 N, L_crumb_* as 37.54, L_crust_* as 41.94, protein as 4.2%, total polyphenols as 186.475 mg GAE/100 g DW, Antioxidant activity as 15.5679 μmol TE/100 g DW, and overall acceptability as 8.2.

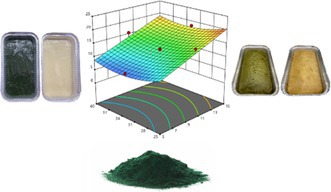

## 1 Introduction

The vegan diet is adopted as a result of ethical principles regarding animal rights, spiritual and religious beliefs, and socioeconomic and environmental concerns (Sebastiani et al. 2019). The number of vegan individuals has increased by 350% in the last decade. In a vegan diet, no products of animal origin are consumed. Generally, nutrients are obtained from the lower levels of the food pyramid (Sakkas et al. 2020). Therefore, the intake of vegetables and fruits, legumes, nuts, and seeds in a vegan diet is high, while the amount of saturated fat taken into the body is lower (Bakaloudi et al. 2021). However, if a balanced nutritional model cannot be created in a vegan diet, it may cause deficiencies in protein, fatty acids, vitamins, and minerals (Marrone et al. 2021).

In today's eating habits, the interest in bakery products such as cakes, bread, and biscuits is increasing day by day. In most parts of the world, cakes are the most consumed cereal products after biscuits and bread (Köten 2021). The cake is a bakery product containing different formulations such as wheat flour, eggs, oil, and sugar. Egg, one of the major ingredients of the cake, contributes to the nutritional value of the cake (Saraç et al. 2022). It also has functions on foods such as creating foam, controlling sugar crystallization, improving color and aroma properties, coagulating, and emulsifying (Yazici and Ozer 2021). The gelling property of eggs is very important in giving volume and textural properties to cakes (Lin et al. 2017). The interest in vegan bakery products has also increased with the increasing vegan diet worldwide. The need for additional structural and nutritional resources to replace these products has increased with no animal products such as milk and eggs being consumed (Saraç et al. 2022). Thus, there has been an increase in the demand for alternative food sources with high functional properties and nutritional value (Pimentel et al. 2021).

According to new health and food trends, demands for food that is environmentally sustainable and enriches daily nutrition are increasing over time (Gün et al. 2022). Due to the rich bioactive components and high protein content of algae, they have the potential to meet the nutritional needs of the increasing population (Koyande et al. 2019). These photosynthetic organisms, which make up approximately 95% of the marine flora, are classified into two groups: microalgae and macroalgae (Sharma and Sharma 2017; Ariede et al. 2017). Interest in microalgae is increasing because of their low‐cost cultivation conditions, rapidly increasing volumes, and various commercial and technological advantages (Silva et al. 2021).

*Spirulina platensis* is a blue‐green microalgae approved by the European Food Safety Authority (EFSA) and the Food and Drug Administration (FDA) (Soni et al. 2017; European Commission 2023). It is among the main foods that can be grown during long‐term space travel, as stated by the European Space Agency and the National Aeronautics and Space Administration (NASA) (Fais et al. 2022). It is defined as the ideal food of the future by the United Nations Educational, Scientific and Cultural Organization (UNESCO); likewise, it is stated as the best health product of humanity by the World Health Organization (WHO) (Soni et al. 2017; Jung et al. 2019). *Spirulina* is rich in proteins (60%–70%), vitamins (especially vitamins E, A, and B), minerals (iron, calcium, zinc, potassium, etc.), essential fatty acids (γ‐linolenic acid, omega‐3, omega‐6), glycolipids, phenolic substances, and pigment substances (such as chlorophylls, phycocyanin, phycoerythrin, and allophycocyanin) (da Silva et al. 2021; Matufi and Choopani 2020; Ragusa et al. 2021; Trotta et al. 2022). This food has a high nutritional content and beneficial effects on health, such as antibacterial, antidiabetic, antiviral, antioxidant, anticancer, antihypertensive, and immunomodulatory (Hu et al. 2019; Jung et al. 2019; Reboleira et al. 2019; Costa et al. 2019).

Microalgae have differences in color, consistency, quality, and nutritional content. They are also included in different food formulations to increase nutritional value. Different microalgae biomass can affect the sensory and physical properties of various bakery products, such as cookies, pasta, and crackers (Fradinho et al. 2020; Pereira et al. 2024). There are several studies in the literature that aim to enrich different foods with *Spirulina* due to its nutritional content and bioactive components (Hassanzadeh et al. 2022; Çelekli et al. 2019). However, there are limited studies on its use in cake formulation (Kim et al. 2008; Barzegar et al. 2021; Güroy 2019). Additionally, there are some studies in the literature on optimizing food formulations enriched with *Spirulina platensis* using Response Surface Methodology (RSM) (Tork et al. 2022; Winarni Agustini et al. 2016; Sengupta and Bhowal 2017). Studies on formulation optimization for bakery products are limited. Gün et al. (2022) optimized the biscuit formulation by including *Spirulina platensis*. Selmo and Salas‐Mellado (2014) optimized the formulation to determine the amounts of methylcellulose, transglutaminase, and *Spirulina* in the rice flour bread formulation. Pagnussatt et al. (2014) aimed to characterize the formulation of partial replacement of pasta prepared with wheat flour with oatmeal, and *Spirulina platensis*. As far as we know, no scientific studies on the production of enriched vegan cakes with *Spirulina* or on the optimization of the formulation for this vegan cake have been published.

Considering recent dietary preferences, the main objectives of this study are the development and optimization of vegan cake formulations enriched with *Spirulina platensis*, rich in bioactive ingredients and protein content. This study aims to expand the range of fortified and sensory‐acceptable baked goods for vegan individuals.

## 2 Materials and Methods

### 2.1 Materials

*Spirulina* (*Spirulina platensis*) used in this study was purchased from a local producer (Algolina Healthy Food Industry Ltd Co.) in Izmir. Wheat flour (Sinangil, Turkey), sugar (Migros, Turkey), sunflower oil (Yudum, Turkey), baking powder (Pakmaya, Turkey), and vanilla powder (Dr. Oetker) were obtained from local markets. Also, xanthan gum was provided by Biokim & Wenda.

### 2.2 Experimental Design

Response surface methodology (RSM) was utilized to create the experimental design and determine the optimal *Spirulina*‐sugar‐flour‐fat levels for a vegan cake made with *Spirulina*. The face‐central composite design (FCCD) was obtained using version 13.0 of the Design‐Expert software (Stat‐Ease Co., Minneapolis, MN, USA). There were 30 experimental runs in this design. Each of the experiments was conducted at the central point with 5 replications. To determine the optimum vegan cake formulation content shown in Table 1, the independent variables were chosen as *Spirulina* content (X_1_: 5, 10, and 15 g), sugar content (X_2_: 90, 100 and 110 g), flour content (X_3_: 90, 100, and 110 g) and fat content (X_4_: 25, 32.5, and 40 g). The dependent variables presented in Table 1 were baking loss, hardness, color (L_crumb_* and L_crust_* values), protein content, total phenolic content, antioxidant activity, and overall acceptability, which affect the nutritional content and quality of the vegan cake enriched with *Spirulina*. The data for the vegan cake samples were replicated in triplicate, and the results were averaged.

**TABLE 1:** Face‐central composite design (FCCD) with variables of formulation (coded and not coded) and experimental results of their responses.

Run	Variables				Responses^a^							
	X_1_	X_2_	X_3_	X_4_	Y_1_	Y_2_	Y_3_	Y_4_	Y_5_	Y_6_	Y_7_	Y_8_
8	5 (−1)	90 (−1)	90 (−1)	25 (−1)	13.04	10.89	33.78	35.57	2.63	179.08	7.72	4.40
20	15 (1)	90 (−1)	90 (−1)	25 (−1)	12.84	40.29	26.33	30.14	4.74	273.37	21.31	5.68
10	5 (−1)	110 (1)	90 (−1)	25 (−1)	12.23	14.24	39.93	40.14	2.67	126.31	7.10	7.24
18	15 (1)	110 (1)	90 (−1)	25 (−1)	11.75	41.93	29.18	32.46	3.73	185.92	17.32	7.08
16	5 (−1)	90 (−1)	110 (1)	25 (−1)	12.19	36.17	40.95	42.16	2.94	180.74	7.41	7.20
13	15 (1)	90 (−1)	110 (1)	25 (−1)	11.99	50.76	31.43	34.89	4.87	258.30	19.53	6.48
12	5 (−1)	110 (1)	110 (1)	25 (−1)	11.41	31.99	47.48	45.42	2.74	153.82	6.80	7.68
2	15 (1)	110 (1)	110 (1)	25 (−1)	10.67	58.27	34.12	43.23	4.28	140.73	16.45	8.40
4	5 (−1)	90 (−1)	90 (−1)	40 (1)	12.84	23.51	34.81	37.85	2.55	159.00	7.74	3.00
25	15 (1)	90 (−1)	90 (−1)	40 (1)	12.99	37.84	25.28	33.16	4.06	171.90	17.50	7.36
17	5 (−1)	110 (1)	90 (−1)	40 (1)	11.42	15.83	41.28	41.60	2.48	131.94	6.61	6.04
7	15 (1)	110 (1)	90 (−1)	40 (1)	11.27	32.53	27.47	33.84	3.90	147.02	15.57	6.92
30	5 (−1)	90 (−1)	110 (1)	40 (1)	11.96	36.85	39.97	44.06	3.06	139.43	6.69	5.76
23	15 (1)	90 (−1)	110 (1)	40 (1)	10.82	54.39	27.45	35.00	5.01	165.13	16.03	7.08
28	5 (−1)	110 (1)	110 (1)	40 (1)	10.51	25.91	39.79	52.63	2.93	144.75	6.41	6.76
15	15 (1)	110 (1)	110 (1)	40 (1)	10.13	53.87	32.49	39.48	4.78	132.37	15.49	8.76
5	5 (−1)	100 (0)	100 (0)	32.5 (0)	12.34	38.58	36.52	41.10	2.72	157.36	8.19	6.03
6	15 (1)	100 (0)	100 (0)	32.5 (0)	11.79	33.13	26.40	33.58	4.34	138.22	18.33	8.48
3	10 (0)	90 (−1)	100 (0)	32.5 (0)	13.78	38.40	29.55	34.60	3.15	134.39	13.08	6.00
27	10 (0)	110 (1)	100 (0)	32.5 (0)	11.29	22.80	32.14	42.98	3.25	85.06	10.83	7.56
11	10 (0)	100 (0)	90 (−1)	32.5 (0)	12.59	21.43	28.51	32.65	3.33	152.20	10.54	5.72
22	10 (0)	100 (0)	110 (1)	32.5 (0)	11.13	30.66	31.80	39.68	3.92	146.66	12.25	6.96
21	10 (0)	100 (0)	100 (0)	25 (−1)	12.21	35.23	31.83	35.91	3.98	159.99	17.01	7.64
24	10 (0)	100 (0)	100 (0)	40 (1)	10.71	33.17	31.24	36.01	3.43	148.97	15.58	7.04
19	10 (0)	100 (0)	100 (0)	32.5 (0)	11.51	35.15	32.78	36.57	3.59	140.73	13.02	6.68
9	10 (0)	100 (0)	100 (0)	32.5 (0)	12.37	38.18	30.97	36.58	3.08	139.76	11.21	6.88
14	10 (0)	100 (0)	100 (0)	32.5 (0)	11.66	28.72	29.82	36.86	2.58	136.19	12.76	7.40
29	10 (0)	100 (0)	100 (0)	32.5 (0)	12.04	27.79	30.01	35.68	2.78	142.52	12.03	8.00
26	10 (0)	100 (0)	100 (0)	32.5 (0)	11.66	30.18	29.94	35.00	3.37	168.92	11.66	6.84
1	10 (0)	100 (0)	100 (0)	32.5 (0)	11.99	28.88	31.91	40.05	3.09	150.90	11.87	7.12

*Note:* X_1_ *Spirulina* (g), X_2_ sugar (g), X_3_ flour (g), X_4_ oil (g), Y_1_ baking loss (%), Y_2_ hardness (N), Y_3_ L_crumb_*, Y_4_ L_crust_*, Y_5_ protein content (%), Y_6_ total phenolic content (mg GAE/100 g DW), Y_7_ antioxidant activity (ABTS) (μmol TE/100 g DW), Y_8_ overall acceptability.

^a^ Mean of triplicate results.

Analysis of variance (ANOVA) was used to determine the adequacy of the model based on the coefficient of determination (*R* ^2^), the lack of fit at the 95% confidence level, and Fisher's test value (*F*‐value). Simultaneously, term reduction was performed on non‐significant effects without disturbing the model hierarchy. The response surface methodology was constructed by plotting 3D plots against four independent variables. The amounts of *Spirulina*, sugar, flour, and oil for the optimum formulation were determined according to the desirability function method, which simultaneously provides minimum hardness, maximum color (L_crumb_* and L_crust_*), maximum protein content, maximum total phenolic content, maximum antioxidant activity, and maximum overall acceptability. The following model was described: the regression analysis used to estimate the dependent variables (Equation 1).

(1) $$Y=\beta_{0}+\sum_{i=1}^{3}\beta_{i}X_{i}+\sum_{i=1}^{3}\beta_{\mathrm{ii}}X_{i}^{2}+\sum_{i=1}^{2}\sum_{j=i+1}^{3}\beta_{\mathrm{ij}}X_{i}X_{j}+\varepsilon$$

where *Y* is the estimated dependent variable, *β*_*0*_ is the constant, *β*_*i*_, *β*_*ii*_, and *β*_*ij*_ are the linear, quadratic, and interaction coefficient regression terms, respectively. Also, *ε* is the error, and both *X*_*i*_ and *X*_*j*_ are the independent variables. To verify the optimization, an independent sample t‐test was conducted by the statistical package SPSS (version 26.0).

### 2.3 Vegan Cake Production

Vegan cakes were produced using a modified version of the method developed by Aida et al. (2018). The amounts of gum (0.55 g), baking powder (3.50 g), and vanilla (3.50 g) were kept constant. The amounts of *Spirulina* powder, sugar, flour, and oil in the formulation of the cakes were used in the amounts included in the experimental design (Table 1). In vegan cake production, sugar, oil, and water were mixed using a household mixer (Fakir, Erica 600 W) at maximum speed for 5 min. After that, wheat flour, baking powder, vanilla, and *Spirulina* powder were added and mixed at minimum speed for 5 min. The resulting cake batter was poured into an aluminum cake mold and baked in a preheated domestic oven (Franke, Switzerland) at 175°C for 30 min. Then, the vegan cakes were left at room temperature for 1 h, and the analyses were started.

### 2.4 Some Chemical Characteristics of Vegan Cakes

#### 2.4.1 Total Phenolic Content and Antioxidant Activity

Moisture contents of vegan cakes were measured in a vacuum oven according to AACC (2000). The total phenolic content and antioxidant activity results of the cakes were given in dry matter.

The extracts used for the evaluation of total phenolic content and antioxidant activity in vegan cakes were obtained according to the Garcia‐Salas et al. (2010) modified method. A volume of 8 mL of 80% methanol (v/v) was added to 4 g of cake sample and extracted in a shaking water bath at 50°C for 1 h. This extract was centrifuged (4°C, 15 min, 4000 rpm) and filtered through Whatmann No. 1. The supernatant was completed to 10 mL with 80% methanol.

Total phenolic contents analyses of vegan cakes were performed according to the modified Folin–Ciocalteu method recommended by Singleton and Rossi (1965). Gallic acid standards were prepared at appropriate concentrations and a standard curve was constructed. The total phenolic contents of the samples were estimated as mg gallic acid equivalent (GAE)/100 g dry weight (DW).

ABTS analysis was used to determine the antioxidant activities of vegan cakes, and the modified method of Re et al. (1999) was applied. The free radical reducing power of the samples will be determined by the TEAC (Trolox equivalent antioxidant capacity) method. ABTS values of the samples were calculated as mg Trolox equivalent (TE)/100 g dry weight (DW) using the % inhibition of the samples and Trolox standard values.

#### 2.4.2 Protein Content

The protein content of vegan cakes was determined by the Dumas method, AACC method 46.30 ( 1995). A conversion factor of 6.25 was used.

### 2.5 Some Physical Characteristics of Vegan Cakes

#### 2.5.1 Baking Loss

Baking loss was calculated according to the method of Martínez‐Cervera et al. (2015). The weight of the cake batter before baking and the weight of the baked and cooled cake for 1 h were weighed. The difference between the weight of the cake batter before baking and the weight of the cake after baking was divided by the weight of the cake batter to calculate the % baking loss.

#### 2.5.2 Color Analysis

The crust and crumb color of the vegan cakes were measured using a Konica Minolta colorimeter (Model CR‐400, Japan). The crust and crumb color parameters of the samples were expressed as L_crust_* and L_crumb_* values for brightness.

#### 2.5.3 Texture Analysis

Texture profile analyses (TPA) of vegan cakes were conducted with a TA‐XT Plus Texture Analyzer (Stable Micro Systems LTD., Godalming, UK). The cake samples were cut 1.5 cm thick to prepare them for texture analysis. The modified method of Dadalı and Elmacı (2019) was applied using a 50 kg load cell and a 36 mm diameter (P/36) cylindrical probe. The hardness values obtained from the TPA curves are given in Newtons (N).

### 2.6 Some Sensory Characteristics of Vegan Cakes

Sensory analysis of vegan cakes was performed with the participation of 50 panelists. The quality criteria of the cake samples were texture, flavor, color, and overall acceptability (Onoğur and Elmacı 2014). Sensory evaluation was applied using a hedonic scale (1–9) (1: I didn't like it at all, 9: I liked it a lot), and overall acceptability values could be modeled using RSM.

## 3 Result and Discussion

The effects of *Spirulina* amount, sugar amount, flour amount, and fat amount on the physical properties, texture properties, chemical properties, and sensory properties of the vegan cake formulation enriched with *Spirulina* are given in Table 2.

**TABLE 2:** Analysis of variance (ANOVA) of the predicted models of baking loss (Y_1_), hardness (Y_2_), L_crumb_* (Y_3_), L_crust_* (Y_4_), protein content (Y_5_), total phenolic content (Y_6_), antioxidant activity (ABTS) (Y_7_), overall acceptability (Y_8_), and variables (X_1_, X_2_, X_3_, and X_4_).

	Y_1_	Y_2_	Y_3_	Y_4_	Y_5_	Y_6_	Y_7_	Y_8_
Model	< 0.0001	< 0.0001	< 0.0001	< 0.0001	< 0.0001	< 0.0001	< 0.0001	< 0.0001
X_1_	0.021	< 0.0001	< 0.0001	< 0.0001	< 0.0001	0,0053	< 0.0001	< 0.0001
X_2_	< 0.0001	—	< 0.0001	< 0.0001	—	< 0.0001	0,0088	< 0.0001
X_3_	< 0.0001	< 0.0001	< 0.0001	< 0.0001	0.0028	—	—	—
X_4_	0.0009	—	—	—	—	0,0005	0,0166	—
X_1_ X_2_	—	—	—	—	—	0,0386	—	—
X_1_ X_3_	—	—	—	—	—	—	—	—
X_1_ X_4_	—	—	—	—	—	0,0244	—	—
X_2_ X_3_	—	—	—	—	—	—	—	—
X_2_ X_4_	—	—	—	—	—	0,0104	—	—
X_3_ X_4_	—	—	0.0176	—	—	—	—	—
X_1_^2^	—	—	0.018	—	—	—	—	—
X_2_^2^	0.0255	—	—	0.0038	—	—	0,0021	—
X_3_^2^	—	—	—	—	0.0151	—	—	—
X_4_^2^	0.0036	—	0.0142	—	—	0,0011	0,0029	—
Lack of fit	0.4149	0.1663	0.4156	0.4763	0.7608	0,1373	0,0812	0.0828
*R* ^2^	0.8576	0.7238	0.9495	0.8695	0.8445	0.7984	0.9383	0.7311
Adjusted *R* ^2^	0.8204	0.7033	0.9335	0.8487	0.8266	0.7342	0.9255	0.7001
Predicted *R* ^2^	0.7255	0.6623	0.8964	0.8	0.7984	0.625	0.8912	0.6005
CV%	2.99	18.33	4.13	4.88	9.17	11.71	9.56	12.14
Standard deviation	0.3536	6.16	1.35	1.85	0.3178	18.32	1.19	0.8465
Mean	11.84	33.59	32.84	37.96	3.47	156.39	12.47	6.97

Model equations were created with linear or quadratic dependent and independent variables. Model coefficients, *p* values, and lack of fit values were determined to characterize the degree of fit of the model. Regression models were effective in predicting the effects of independent variables on responses. The responses, including baking loss, L_crust_*, L_crumb_*, protein content, total phenolic content, and ABTS antioxidant activity value, were described by a reduced quadratic model, while the responses such as hardness and overall acceptability were characterized by a reduced linear model. The reduced linear and reduced quadratic model equations for the variables are given in Table 3. ANOVA was applied to determine the linear and quadratic effects of *Spirulina* amount, sugar amount, flour amount, and fat amount on vegan cake properties. The statistical significance of the model, variables and their interactions, model coefficients, and lack of fit are given in Table 2.

**TABLE 3:** Final equations in terms of coded factors.

Responses	Equation
Protein	3.28 + 0.83X_1_ + 0.25X_3_ + 0.31 $X_{3}^{2}$
Hardness	33.59 + 9.39X_1_ + 7.8X_3_
Baking loss (%)	11.93–0.21X_1_–0.65X_2_–0.56X_3_–0.32X_4_ + 0.46 $X_{2}^{2}$ – 0.62 $X_{4}^{2}$
L_crumb_*	30.55–5.24X_1_ + 1.91X_2_ + 2.16X_3_–0.85X_4_–0.87X_3_X_4_ + 1.87 $X_{2}^{2}$ + 1.95 $X_{4}^{2}$
L_crust_*	36.64–3.6X_1_ + 2.46X_2_ + 3.29X_3_ + 2.27 $X_{2}^{2}$
Total phenolic content	141.08 + 13.36X_1_–22.97X_2_–17.65X_4_–10.08X_1_X_2_–11.07X_1_X_4_ + 12.83X_2_X_4_ + 25.52 $X_{4}^{2}$
ABTS antioxidant capacity	12.52 + 5.16 X_1_–0.8009X_2_–0.7237X_4_–2.21 $X_{2}^{2}$ + 2.13 $X_{4}^{2}$
Overall acceptability	6.81 + 0.6739X_1_ + 0.7489X_2_ + 0.6467X_3_

The calculated model values of each response were statistically significant at a 95% confidence interval (*p* < 0.05). The *F*‐values obtained with Fisher's test showed low *p* values for all independent variables (*p* < 0.05). Lack of fit indicates the success of the model and should be *p* > 0.05. In this study, the lack of fit values of all responses were found to be insignificant (*p* > 0.05). As seen in Table 2, it was determined that the experimental data had good reproducibility. In addition, the % coefficient of variation (CV %) and Adeq. Precision values also support model adequacy. The *R* ^2^ values expressing the percent change of the model responses varied between 0.7238 and 0.9495 (Table 2).

### 3.1 Effect of Variables on Some Chemical Characteristics of Vegan Cakes

**FIGURE 1:**
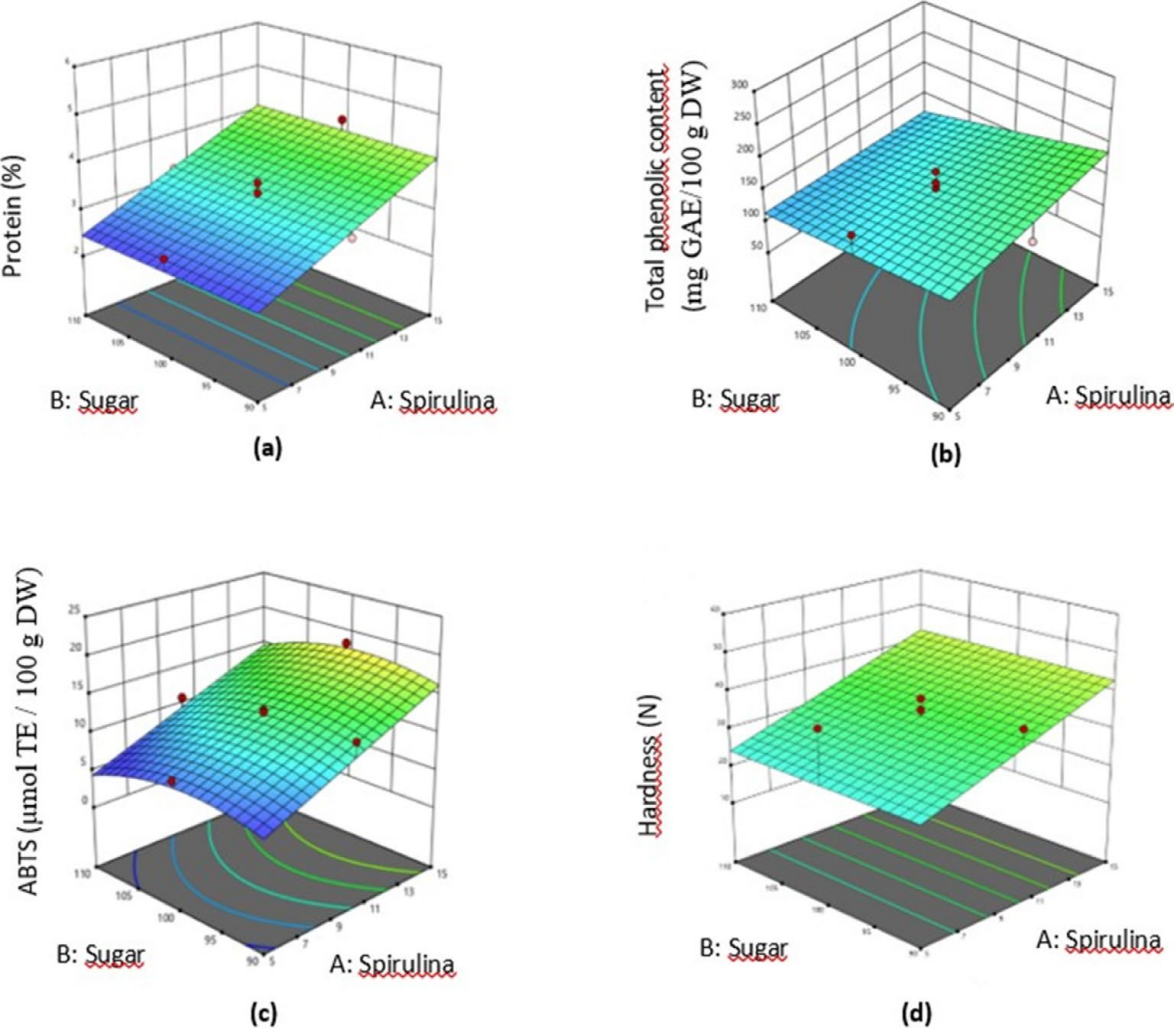
Response surface 3D plots indicating interaction effects of both sugar and *Spirulina* content on protein content (a), total phenolic content (b), ABTS antioxidant capacity (c), and hardness (d).

As shown in Table 1, the protein contents of vegan cakes enriched with *Spirulina* ranged from 2.48% to 5.01%. The effects of independent variables on % protein contents were elucidated by a reduced quadratic model. The amount of *Spirulina* (X_1_), the amount of flour (X_3_), and the quadratic effect of flour (X_3_^2^) had a statistically significant effect (*p* < 0.05) (Table 2). The *p* value of 0.7608 in the lack of fit test indicates that the lack of fit was insignificant (*p* > 0.05). There was a linear increase in protein content as the amount of *Spirulina* increased (*p* < 0.05). As the flour content of the vegan cake increased, the protein content of the cake first decreased and then slightly increased (*p* < 0.05). The coefficients of the variables in the regression model for the protein content of vegan cakes are given in Table 3. As seen in the 3D response surface graphs in Figure 1a and the Data S1, it was determined that the amount of sugar and fat did not affect the protein contents (*p* > 0.05). Compared to the studies by Güroy (2020), Barakat et al. (2016), Moradi et al. (2024) and Hussein et al. (2021), the protein contents of the vegan cakes obtained in this study were lower. The reason for the lower protein content compared to similar bakery products in other studies is thought to be due to the ingredients used. In this study, eggs and milk were not used because vegan cake was produced. This is thought to affect the protein contents of products. Also, similar to the literature, the results showed that there was a linear relationship between the increase in the amount of *Spirulina* and the increase in protein content.

As shown in Table 1, the TPC contents of vegan cakes enriched with *Spirulina* changed from 85.06 to 258.30 mg GAE/100 g DW, respectively. The effects of independent variables on phenolic content were explained by a reduced quadratic model. The linear effect of *Spirulina* content (X_1_), sugar content (X_2_), fat content (X_4_), the interaction effect of *Spirulina* and sugar (X_1_X_2_), the interaction effect of *Spirulina* and fat (X_1_X_4_), the interaction effect of sugar and fat (X_2_X_4_), and the quadratic effect of fat content (X_4_^2^) were statistically significant (*p* < 0.05) (Table 2). The *p* value of 0.1373 in the lack of fit test shows that the lack of fit was insignificant (*p* > 0.05). There was a linear increase in phenolic content as the amount of *Spirulina* increased (*p* < 0.05). The increase in sugar content caused a linear decrease in phenolic content (*p* < 0.05). The phenolic content decreased quadratically as the fat content of vegan cake increased (*p* < 0.05). The coefficients of the variables in the regression model for the TPC values of vegan cakes are given in Table 3. 3D response surface plots are shown in Figure 1b and the Data S1. In the study by Zlateva et al. (2020), *Spirulina* was included in the bread formulation at 2% and 4% as a flour substitute. They found the phenolic content of the breads to be 0.74 mg GAE/g DW and 0.88 mg GAE/g DW, respectively. Hussein et al. (2021) added 2.5%, 5%, 7.5%, and 10% *Spirulina* in pasta formulation. They found the total phenolic content to be 0.85 mg/g, 1.94 mg/g, 2.16 mg/g, 2.45 mg/g, and 3.12 mg/g before cooking and 0.72 mg/g, 1.56 mg/g, 1.56 mg/g, 1.95 mg/g, 2.13 mg/g, and 2.95 mg/g after cooking, respectively. Saharan and Jood (2021) investigated the use of *Spirulina* powder as a substitute for wheat flour in bread making at 2%, 4%, and 6%. The phenolic contents of the breads were found to be 0.85 mg GAE/g DW, 1.19 mg GAE/g DW, and 1.79 mg GAE/g DW, respectively. Similar to studies in the literature, as the amount of *Spirulina* increased, a directly proportional increase in the total phenolic content of the vegan cakes was observed in this study.

As presented in Table 1, the ABTS values of vegan cakes enriched with *Spirulina* varied from 6.41 to 21.31 μmol TE/100 g DW. The effects of independent variables on the antioxidant activity were described by a reduced quadratic model. The linear effects of *Spirulina* content (X_1_), sugar content (X_2_), and fat content (X_4_), and the quadratic effects of both sugar content (X_2_^2^) and fat content (X_4_^2^) indicated statistically significant effects (*p* < 0.05) (Table 2). The *p* value of 0.0812 in the lack of fit test revealed that the lack of fit was insignificant (*p* > 0.05). There was a linear increase in antioxidant activity as the amount of *Spirulina* increased (*p* < 0.05). Sugar content had a quadratic effect on antioxidant activity (*p* < 0.05). According to the 3D response surface plots in Figure 1c and the Data S1, the antioxidant activity increased initially and then decreased as the sugar content of the vegan cake increased, while it decreased initially and then increased as the fat content of the vegan cake increased (*p* < 0.05). The coefficients of the variables in the regression model for ABTS values of vegan cakes are given in Table 3. As in the studies conducted by Egea et al. (2014), Amoriello et al. (2021) and Peñalver and Nieto (2024), it was found that the antioxidant activity increased in direct proportion to the increase in the amount of *Spirulina* in this study.

### 3.2 Effect of Variables on Some Physical Characteristics of Vegan Cakes

Hardness represents the highest force measured at the first compression. The hardness value has a linear relationship with protein in the product, while there is an inverse relationship with fat and moisture content (Chueamchaitrakun et al. 2011; Marcet et al. 2015). As seen in Table 1, the hardness of the vegan cakes enriched with *Spirulina* changed from 10.89 N to 58.27 N. The effects of the independent variables on the hardness of vegan cakes accounted for a reduced linear model. Both the linear terms of *Spirulina* (X_1_) and flour (X_3_) amounts had statistically significant effects (*p* < 0.05) (Table 2). The *p* value found to be 0.1663 in the lack of fit test revealed that the lack of fit was insignificant (*p* > 0.05). There was a linear increase in the hardness of the vegan cake as the amount of *Spirulina* increased, and increasing the amount of flour also had a linear effect on the hardness of the vegan cake (*p* < 0.05). The coefficients of the variables in the regression model for the hardness of vegan cakes are given in Table 3. As seen in the 3D response surface graphs in Figure 1d and Data S1, it was found that the amount of sugar and fat had no effect on the hardness of the vegan cake (*p* > 0.05). Sugar crystallizes after cooling and has a harder structure. Thus, as the amount of sugar in the cake increases, its hardness also increases (Jan et al. 2018). The results were similar to the study of Marzec et al. (2023), Fanari et al. (2023) and Su et al. (2025), but they were higher than Moradi et al. (2024). It is thought that this difference may be related to the analysis method applied and the ingredients used in the cake formulation.

**FIGURE 2:**
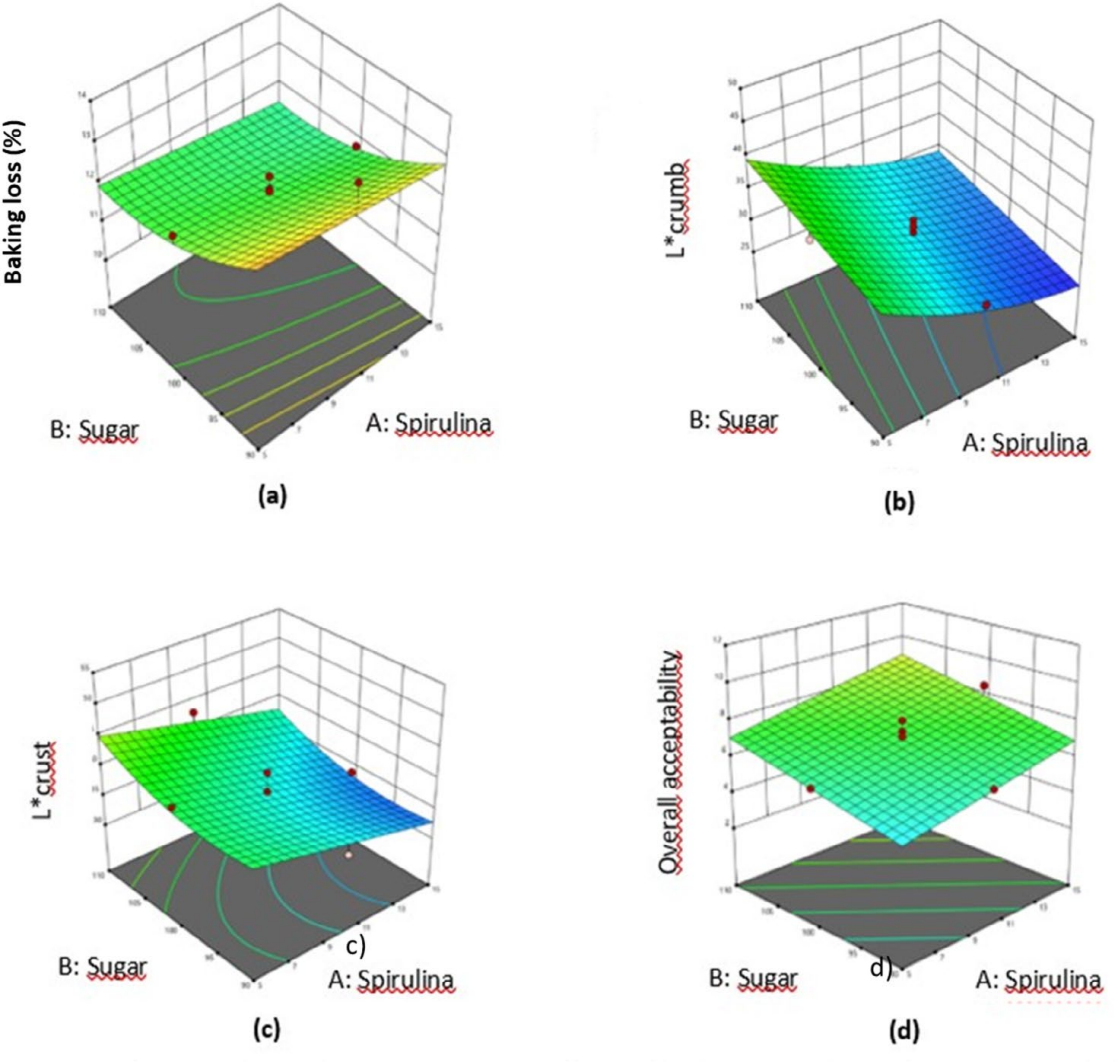
Response surface 3D plots indicating interaction effects of both sugar and *Spirulina* content on baking loss (a), L_crumb_* (b), L_crust_* (c), and overall acceptability (d).

While the minimum baking loss value of vegan cakes enriched with *Spirulina* was 0.13%, their maximum baking loss value was found to be 13.77%. The effects of independent variables on baking loss were expressed by a reduced quadratic model. *Spirulina* amount (X_1_), sugar amount (X_2_), flour amount (X_3_), and fat amount (X_4_), as well as the quadratic effects of sugar amount (X_2_^2^) and fat amount (X_4_^2^) exhibited statistically significant effects (*p* < 0.05) (Table 2). In the lack of fit test, the *p* value was obtained as 0.4149, and this value indicated that the lack of fit was insignificant (*p* > 0.05). Sugar content and fat content had a second‐order effect on baking loss (*p* < 0.05). The baking loss of vegan cakes decreased initially and then increased as the fat content of the vegan cake increased. On the contrary, as the amount of sugar in vegan cakes increased, the baking loss increased initially and then decreased. There was a linear decrease in baking loss as both *Spirulina* and flour contents in vegan cake increased (*p* < 0.05). The coefficients of the variables in the regression model for the baking loss (%) of vegan cakes are shown in Table 3. 3D response surface plots are illustrated in Figure 2a and Data S1. Since no data on baking loss is available in studies of cakes including *Spirulina*, these results were compared with other bakery products. The results are in line with those of Diprat et al. (2020) and El‐Kewaisny et al. (2017). Cake dough is expected to develop symmetrically during baking, and the symmetry index is expected to be higher than zero (Felisberto et al. 2015). The symmetry index of vegan cakes containing *Spirulina* is greater than zero and is thought to be desirable. *Spirulina*'s fiber content, ability to increase viscosity, and water binding capacity prevented more uneven rising, resulting in a more symmetrical cake shape (Çakır et al. 2024). The water‐binding capacity of the dough increased with the addition of *Spirulina* and supported rising by retaining more air bubbles (Onacik‐Gür et al. 2018). These factors may have caused the increase in the symmetry index.

Color values in bakery products generally vary depending on the physicochemical properties of the dough, baking temperature, and time. The color of the cake crust is related to caramelization and Maillard reactions (Aydogdu et al. 2018). The L value is used for lightness/darkness expression (Nakilcioğlu and Ötleş 2022). The L_crumb_* values of vegan cakes enriched with *Spirulina* ranged from 25.28 to 47.48. The effects of the independent variables on L_crumb_* color were explained by a reduced quadratic model. The linear effects of *Spirulina* amount (X_1_), sugar amount (X_2_), flour amount (X_3_), and fat amount (X_4_), the interaction effect of flour and fat (X_3_X_4_), and quadratic effects of *Spirulina* amount (X_1_^2^), and fat amount (X_4_^2^) were found to be statistically significant effects (*p* < 0.05) (Table 2). The *p* value detected in the lack of fit test was 0.4156, and this showed that the lack of fit was insignificant (*p* > 0.05). As the amount of sugar increased, there was a linear increase in L_crumb_* color (*p* < 0.05). The amount of flour also caused a linear increase in L_crumb_* color (*p* < 0.05). The amount of *Spirulina* had a second‐order effect on L_crumb_* color (*p* < 0.05) and L_crumb_* color decreased quadratically with increasing *Spirulina* content in vegan cake. At the same time, the amount of fat had a second‐order effect on the L_crumb_* color (*p* < 0.05). The L_crumb_* color of vegan cakes also decreased slightly at first and then increased sharply as the fat content of the vegan cake increased. The L_crust_* color of vegan cakes enriched with *Spirulina* was between 30.14 and 52.63. The effects of independent variables on L_crust_* color were elucidated by a reduced quadratic model. The linear effects of *Spirulina* content (X_1_), sugar content (X_2_), and flour content (X_3_), as well as the quadratic effect of sugar content (X_2_^2^) were observed to be statistically significant (*p* < 0.05) (Table 2). The *p* value of 0.4763 in the lack of fit test demonstrated that the lack of fit was insignificant (*p* > 0.05). As the amounts of *Spirulina* in vegan cake increased, there was a linear decrease in L_crust_* color (*p* < 0.05). The amount of flour also caused a linear increase in L_crust_* color (*p* < 0.05). Also, L_crust_* color of vegan cake increased quadratically as the sugar content of vegan cake increased (*p* < 0.05). The coefficients of the variables in the regression model for L_crumb_* and L_crust_* colors of vegan cakes are indicated in Table 3. 3D response surface plots are represented in Figure 2b, Figure 2c, and Data S1. The values of L_crumb_* and L_crust_* color were similar to the studies by Su et al. (2025), Moradi et al. (2024), Peñalver and Nieto (2024), Marzec et al. (2023), El‐Said et al. (2021), and Amoriello et al. (2021). Considering these studies in the literature, the L* value of bakery products decreases with an increase in the amount of a blue‐green algae species such as *Spirulina* or Chlorella. In this study, the inclusion of *Spirulina* in the vegan cake formulation caused a decrease in brightness in both L_crumb_* and L_crust_* color. The addition of *Spirulina* to the vegan cake resulted in a darker color on both the inside and outside of the cake. Therefore, the dark color formed on both the inside and outside of the cake by adding *Spirulina* was manifested by a decrease in brightness. This is a result of *Spirulina*'s own green‐blue and low‐brightness color.

### 3.3 Effect of Variables on Some Sensory Properties of Vegan Cakes

As shown in Table 1, the values of overall acceptability related to the vegan cakes enriched with *Spirulina* changed between 3.00 and 8.76. The effects of the independent variables on the overall acceptability value were determined with a reduced linear model. The linear effects of *Spirulina* amount (X_1_), sugar amount (X_2_), and flour amount (X_3_) revealed statistically significant effects (*p* < 0.05) (Table 2). In the lack of fit test, the *p* value was determined to be 0.0828. This value indicated that the lack of fit was insignificant (*p* > 0.05). As the amount of *Spirulina* increased, there was a linear increase in the overall acceptability value (*p* < 0.05). Increasing the amount of sugar caused a linear increase in overall acceptability (*p* < 0.05). Also, the amount of flour affected the overall acceptability in a linearly increasing relationship (*p* < 0.05). The coefficients of the variables in the regression model for the overall acceptability of vegan cakes are given in Table 3. As observed in the 3D response surface graphs in Figure 2d and the Data S1, the amount of fat did not significantly affect the overall acceptability (*p* > 0.05). The obtained overall acceptability values are in the same trend as the values found by Saharan and Jood (2021), Ali (2022), and Hussein et al. (2021). Also, these results were higher than those of Moradi et al. (2024). It is thought that this difference may be due to the composition of the product, *Spirulina* species, its usage ratio, taste, and odor.

### 3.4 Formulation Optimization and Validation of Predicted Models

The optimization of vegan cake formulation enriched with *Spirulina* was determined according to the “desirability function” method to simultaneously maximize L_crumb_*, L_crust_*, protein content, total phenolic content, antioxidant activity, and overall acceptability. Also, the hardness value was expected to be minimal while the value of baking loss was kept within range. The optimum formulation of vegan cake enriched with *Spirulina* obtained as a result of numerical analysis was 11.965 g *Spirulina*, 106.206 g sugar, 110 g flour, and 25 g oil. Vegan cake enriched with *Spirulina* was produced in five replicates using the optimum formulation. The results of the analysis performed on the optimum cakes are presented in Table 4. Statistical analysis was performed to determine any significant difference between the predicted values and the observed values. No significant difference was found (*p* > 0.05). As seen in Table 4, the percentage error values vary between 1.04% and 9.21%. The data obtained in the validation analyses show that the developed model is appropriate. The optimum conditions determined can be recommended for the production of the highest quality vegan cake enriched with *Spirulina* in terms of baking loss, hardness, L_crumb_*, L_crust_*, protein content, total phenolic content, antioxidant activity (ABTS) and overall acceptability.

**TABLE 4:** Criteria and results of the numerical optimization of the responses for the vegan cake enriched with *Spirulina*.

Response	Goal	Predicted value	Experimental value^a^	SE^b^	Difference	% Error^c^	*p*
Y_1_	In range	10.76	11.22 ± 0.22	0.185	0.46	4.14	0.413
Y_2_	Minimize	44.98	43.96 ± 1.13	1.92	1.02	2.32	0.868
Y_3_	Maximize	35.81	37.54 ± 0.39	0.88	1.73	4.62	0.462
Y_4_	Maximize	40.88	41.94 ± 1.37	0.65	1.05	2.51	0.614
Y_5_	Maximize	4.16	4.20 ± 0.19	0.11	0.04	1.04	0.894
Y_6_	Maximize	169.31	186.47 ± 9.84	7.69	10.05	9.21	0.482
Y_7_	Maximize	16.02	15.57 ± 1.16	0.56	2.82	2.93	0.753
Y_8_	Maximize	8.17	8.20 ± 0.34	0.25	0.69	0.37	0.968

*Note:* Y_1_ baking loss (%), Y_2_ hardness (N), Y_3_ L_crumb_*, Y_4_ L_crust_*, Y_5_ protein content (%), Y_6_ total phenolic content (mg GAE/100 g DW), Y_7_ antioxidant activity (ABTS) (μmol TE/100 g DW), Y_8_ overall acceptability.

^a^ Mean ± standard deviation of five replicate determinations.

^b^ Mean standard error.

^c^ % error = (|yexp−ypre|/yexp) × 100.

## 4 Conclusion

RSM has been successfully used to optimize the formulation of vegan cake enriched with *Spirulina*. The amounts of *Spirulina*, sugar, flour, and fat markedly affected the baking loss, hardness value, color parameters including L_crumb_* and L_crust_*, protein content, total phenolic content, antioxidant activity content, and overall acceptability of vegan cakes. The model equation developed can be utilized for predicting some physical, chemical, and sensory quality parameters of vegan cakes enriched with *Spirulina*. Based on the 3D graph and ANOVA results, the main formulation of vegan cake with desired quality could be obtained by incorporating 11.965 g *Spirulina*, 106.206 g sugar, 110 g flour, and 25 g oil. Vegan cake enriched with *Spirulina* can be an alternative food source for individuals with nutritional deficiencies. It can also meet the need for sustainable resources in the vegan food industry. This study will lead to the expansion of functional food alternatives that can be used by vegan individuals and the development of similar products using *Spirulina*.

## Author Contributions

**Eda Nurko:** conceptualization (equal), data curation (equal), formal analysis (lead), funding acquisition (equal), investigation (equal), methodology (equal), project administration (supporting), resources (equal), software (equal), supervision (equal), validation (equal), visualization (equal), writing – original draft (equal), writing – review and editing (equal). **Emine Nakilcioğlu:** conceptualization (equal), data curation (equal), formal analysis (supporting), funding acquisition (equal), investigation (equal), methodology (equal), project administration (lead), resources (equal), software (equal), supervision (equal), validation (equal), visualization (equal), writing – original draft (equal), writing – review and editing (equal).

## Conflicts of Interest

The authors declare no conflicts of interest.

## Supporting information


Data S1.

## Data Availability

The data that support the findings of this study are available from the corresponding author upon reasonable request.

## References

[fsn370116-bib-0001] AACC . 1995. “Crude Protein—Combustion Method, 46–30.01.” Approved Methods of the AACC. St. Paul, USA.

[fsn370116-bib-0065] AACC . 2000. "Approved methods of the AACC (10th ed)". American Association of Cereal Chemists, St. Paul.

[fsn370116-bib-0002] Aida, F. M. N. A. , A. Z. Nor Anida , and Z. Norasmanizan . 2018. “A Study on Physicochemical and Sensory Characteristics of Eggless Yellow Cake.” Indonesian Food Science and Technology Journal 2, no. 1: 1–8.

[fsn370116-bib-0069] Ali H . 2022. “Nutritional Value, Amino Acids of Biscuits and Cakes Fortified With Spirulina (Arthrospira platensis) Powder.” Journal of Home Economics, 23, no. 4: 141–158.

[fsn370116-bib-0003] Amoriello, T. , F. Mellara , M. Amoriello , D. Ceccarelli , and R. Ciccoritti . 2021. “Powdered Seaweeds as a Valuable Ingredient for Functional Breads.” European Food Research and Technology 247, no. 10: 2431–2443.

[fsn370116-bib-0004] Ariede, M. B. , T. M. Candido , A. L. M. Jacome , M. V. R. Velasco , J. C. M. Carvalho , and A. R. Baby . 2017. “Cosmetic Attributes of Algae—A Review.” Algal Research 25: 483–487.

[fsn370116-bib-0005] Aydogdu, A. , G. Sumnu , and S. Sahin . 2018. “Effects of Addition of Different Fibers on Rheological Characteristics of Cake Batter and Quality of Cakes.” Journal of Food Science and Technology 55: 667. 29391631 10.1007/s13197-017-2976-y PMC5785392

[fsn370116-bib-0006] Bakaloudi, D. R. , A. Halloran , H. L. Rippin , et al. 2021. “Intake and Adequacy of the Vegan Diet: A Systematic Review of the Evidence.” Clinical Nutrition 40, no. 5: 3503–3521. 33341313 10.1016/j.clnu.2020.11.035

[fsn370116-bib-0007] Barakat, E. H. , N. M. El‐Kewaisny , and A. A. Salama . 2016. “Chemical and Nutritional Evaluation of Fortified Biscuits With Dried *Spirulina* Algae.” Journal of Food and Dairy Sciences 7, no. 3: 167–177.

[fsn370116-bib-0008] Barzegar, H. , N. Zangene , and E. Abdolnabipoor . 2021. “Effect of *Spirulina platensis* Microalgae Powder as an Egg White Substitute on the Sponge Cake Properties.” Journal of Food Science and Technology 17, no. 108: 31–44.

[fsn370116-bib-0009] Çakır, E. , H. Bekiroğlu , B. Göker , and O. Sağdıç . 2024. “Effect of *Spirulina platensis* on Textural, Sensory and Some Physicochemical Characteristics in Gluten‐Free Functional Biscuit Production.” Çukurova Tarım Ve Gıda Bilimleri Dergisi 39, no. 1: 82–96.

[fsn370116-bib-0010] Çelekli, A. , Z. A. Alslibi , and H. Üseyin Bozkurt . 2019. “Influence of Incorporated *Spirulina platensis* on the Growth of Microflora and Physicochemical Properties of Ayran as a Functional Food.” Algal Research 44: 101710.

[fsn370116-bib-0011] Chueamchaitrakun, P. , P. Chompreeda , V. Haruthaithanasan , T. Suwonsichon , S. Kasemsamran , and W. Prinyawiwatkul . 2011. “Sensory Descriptive and Texture Profile Analyses of Butter Cakes Made From Composite Rice Flours.” International Journal of Food Science and Technology 46: 2358–2365.

[fsn370116-bib-0012] Costa, J. A. V. , B. C. B. Freitas , G. M. Rosa , L. Moraes , M. G. Morais , and B. G. Mitchell . 2019. “Operational and Economic Aspects of *Spirulina*‐Based Biorefinery.” Bioresource Technology 292: 121946. 31422868 10.1016/j.biortech.2019.121946

[fsn370116-bib-0013] Dadalı, C. , and Y. Elmacı . 2019. “Reduction of Sucrose by Inhomogeneous Distribution in Cake Formulation.” Journal of Food Measurement and Characterization 13, no. 4: 2563–2570.

[fsn370116-bib-0014] Diprat, A. B. , R. C. S. Thys , E. Rodrigues , and R. Rech . 2020. “ *Chlorella sorokiniana*: A New Alternative Source of Carotenoids and Proteins for Gluten‐Free Bread.” Lebensmittel‐Wissenschaft & Technologie 134: 109974.

[fsn370116-bib-0015] Egea, B. C. B. , A. L. M. Campos , J. C. M. De Carvalho‐Eliane , and D. G. Danesi . 2014. “Antioxidant and Nutritional Potential of Cookies Enriched With *Spirulina platensis* and Sources of Fibre.” Journal of Food and Nutrition Research 53, no. 2: 171–179.

[fsn370116-bib-0016] El‐Kewaisny, N. , S. El‐Baset , and B. Ekram . 2017. “Organoleptic and Chemical Properties of Biscuits Blends Lipids Supported With Dried *Spirulina* Algae During Storage.” Journal of Specific Education and Technology, Scientific and Applied Research 4, no. 1: 753–769.

[fsn370116-bib-0068] El‐Said, E. T. , A. S. Soliman , M. S. Abbas , and S. E. Aly . 2021. “Treatment of Anaemia and Malnutrition by Shamy Bread Fortified With Spirulina, Quinoa and Chickpea Flour.” Egyptian Journal of Chemistry 64, no. 5: 2253–2268.

[fsn370116-bib-0017] European Commission . 2023. “European Union, Novel Food Catalogue.” https://ec.europa.eu/food/food‐feed‐portal/screen/novel‐food‐catalogue/search.

[fsn370116-bib-0018] Fais, G. , A. Manca , F. Bolognesi , et al. 2022. “Wide Range Applications of *Spirulina*: From Earth to Space Missions.” Marine Drugs 20, no. 5: 299. 35621951 10.3390/md20050299 PMC9143897

[fsn370116-bib-0019] Fanari, F. , J. Comaposada , F. Boukid , et al. 2023. “Enhancing Energy Bars With Microalgae: A Study on Nutritional, Physicochemical and Sensory Properties.” Journal of Functional Foods 109: 105768.

[fsn370116-bib-0020] Felisberto, M. H. , A. L. Wahanik , C. R. Gomes‐Ruffi , M. T. P. S. Clerici , Y. K. Chang , and C. J. Steel . 2015. “Use of Chia (*Salvia hispanica* L.) Mucilage Gel to Reduce Fat in Pound Cakes.” LWT—Food Science and Technology 63, no. 2: 1049–1055.

[fsn370116-bib-0021] Fradinho, P. , A. Niccolai , R. Soares , et al. 2020. “Effect of *Arthrospira platensis* (*Spirulina*) Incorporation on the Rheological and Bioactive Properties of Gluten‐Free Fresh Pasta.” Algal Research 45: 101743.

[fsn370116-bib-0022] Garcia‐Salas, P. , A. Morales‐Soto , A. Segura‐Carretero , and A. Fernández Gutiérrez . 2010. “Phenolic Compound Extraction Systems for Fruit and Vegetable Samples.” Molecules 15, no. 12: 8813–8826. 21131901 10.3390/molecules15128813 PMC6259353

[fsn370116-bib-0023] Gün, D. , A. Çelekli , H. Bozkurt , and S. Kaya . 2022. “Optimization of Biscuit Enrichment With the Incorporation of *Arthrospira platensis*: Nutritional and Sensory Approach.” Journal of Applied Phycology 34, no. 3: 1555–1563.

[fsn370116-bib-0024] Güroy, B. 2019. “Determination of the Phycocyanin, Protein Content and Sensory Properties of Muffins Containing *Spirulina* Powder or Fresh *Spirulina* .” Journal of Food Feed Science and Technology 23: 10–18.

[fsn370116-bib-0066] Güroy, B . 2020. “Determination of the Phycocyanin, Protein Content and Sensory Properties of Mufﬁns Containing Spirulina Powder or Fresh Spirulina.” Journal of Food and Feed Science‐Technology 23:10–18.

[fsn370116-bib-0025] Hassanzadeh, H. , B. Ghanbarzadeh , Y. Galali , and H. Bagheri . 2022. “The Physicochemical Properties of the *Spirulina*‐Wheat Germ‐Enriched High‐Protein Functional Beverage Based on Pear‐Cantaloupe Juice.” Food Science & Nutrition 10, no. 11: 3651–3661. 36348790 10.1002/fsn3.2963 PMC9632204

[fsn370116-bib-0026] Hu, S. , X. Fan , P. Qi , and X. Zhang . 2019. “Identification of Anti‐Diabetes Peptides From *Spirulina platensis* .” Journal of Functional Foods 56: 333–341.

[fsn370116-bib-0027] Hussein, A. , G. Ibrahim , M. Kamil , M. El‐Shamarka , S. Mostafa , and D. Mohamed . 2021. “ *Spirulina*‐Enriched Pasta as Functional Food Rich in Protein and Antioxidant.” Biointerface Research in Applied Chemistry 11: 14736–14750.

[fsn370116-bib-0028] Jan, K. N. , P. S. Panesar , and S. Singh . 2018. “Optimization of Antioxidant Activity, Textural and Sensory Characteristics of Gluten‐Free Cookies Made From Whole Indian Quinoa Flour.” LWT—Food Science and Technology 93: 573–582.

[fsn370116-bib-0029] Jung, F. , A. Krüger‐Genge , P. Waldeck , and J. H. Küpper . 2019. “ *Spirulina platensis*, a Super Food?” Journal of Cellular Biotechnology 5, no. 1: 43–54.

[fsn370116-bib-0030] Kim, M. H. , H. J. Kim , M. Y. Kim , and M. R. Kim . 2008. “Optimization of *Spirulina* Madeleine Using Response Surface Methodology.” Journal of the Korean Society of Food Culture 23, no. 6: 761–770.

[fsn370116-bib-0031] Köten, M. 2021. “Influence of Raw/Roasted Terebinth ( *Pistacia terebinthus* L.) on the Selected Quality Characteristics of Sponge Cakes.” International Journal of Gastronomy and Food Science 24: 100342.

[fsn370116-bib-0032] Koyande, A. K. , K. Chew , K. Rambabu , Y. Tao , D. T. Chu , and P. L. Show . 2019. “Microalgae: A Potential Alternative to Health Supplementation for Humans.” Food Science and Human Wellness 8, no. 1: 16–24.

[fsn370116-bib-0033] Lin, M. , S. Tay , H. Yang , B. Yang , and H. Li . 2017. “Development of Eggless Cakes Suitable for Lacto‐Vegetarians Using Isolated Pea Proteins.” Food Hydrocolloids 69: 440–449.

[fsn370116-bib-0034] Marcet, I. , B. Paredes , and M. Díaz . 2015. “Egg Yolk Granules as Low‐Cholesterol Replacer of Whole Egg Yolk in the Preparation of Gluten‐Free Muffins.” Food Science and Technology 62, no. 1: 613–619.

[fsn370116-bib-0035] Marrone, G. , C. Guerriero , D. Palazzetti , et al. 2021. “Vegan Diet Health Benefits in Metabolic Syndrome.” Nutrients 13, no. 3: 817. 33801269 10.3390/nu13030817 PMC7999488

[fsn370116-bib-0036] Martínez‐Cervera, S. , A. Salvador , and T. Sanz . 2015. “Cellulose Ether Emulsions as Fat Replacers in Muffins: Rheological, Thermal and Textural Properties.” LWT—Food Science and Technology 63: 1083–1090.

[fsn370116-bib-0037] Marzec, A. , P. Kramarczuk , H. Kowalska , and J. Kowalska . 2023. “Effect of Type of Flour and Microalgae ( *Chlorella vulgaris* ) on the Rheological, Microstructural, Textural, and Sensory Properties of Vegan Muffins.” Applied Sciences 13, no. 13: 7632.

[fsn370116-bib-0038] Matufi, F. , and A. Choopani . 2020. “ *Spirulina*, Food of Past, Present and Future.” Health Biotechnology and Biopharma 3, no. 4: 1–20.

[fsn370116-bib-0039] Moradi, Y. , M. Ghaeni , and H. Hadaegh . 2024. “Comparison of the Effect of Adding *Spirulina platensis* Powder on Sensory, Physical, Protein and Iron Properties of Three Different Industrial Products of Bread, Cake and Layered Sweets.” Iranian Food Science and Technology Research Journal 20, no. 1: 153–164.

[fsn370116-bib-0040] Nakilcioğlu, E. , and S. Ötleş . 2022. “Multiresponse Optimization of Physical, Chemical, and Sensory Properties of the Gluten‐Free Cake Made With Whole White Quinoa Flour.” Journal of Food Science and Technology 59, no. 10: 3836–3847. 36193384 10.1007/s13197-022-05406-3 PMC9525520

[fsn370116-bib-0041] Onacik‐Gür, S. , A. Żbikowska , and B. Majewska . 2018. “Effect of *Spirulina* (*Spirulina platensis*) Addition on Textural and Quality Properties of Cookies.” Italian Journal of Food Science 30, no. 1: 1–12.

[fsn370116-bib-0042] Onoğur, T. , and Y. Elmacı . 2014. Gıdalarda Duyusal Değerlendirme. Sidas Medya Ltd. Şti.

[fsn370116-bib-0043] Pagnussatt, F. A. , F. Spier , T. E. Bertolin , J. A. V. Costa , and L. C. Gutkoski . 2014. “Technological and Nutritional Assessment of Dry Pasta With Oatmeal and the Microalga *Spirulina platensis* .” Brazilian Journal of Food Technology 17: 296–304.

[fsn370116-bib-0044] Peñalver, R. , and G. Nieto . 2024. “Developing a Functional Gluten‐Free Sourdough Bread by Incorporating Quinoa, Amaranth, Rice and *Spirulina* .” LWT—Food Science and Technology 201: 116162.

[fsn370116-bib-0045] Pereira, T. , S. Costa , S. Barroso , P. Teixeira , S. Mendes , and M. M. Gil . 2024. “Development and Optimization of High‐Protein and Low‐Saturated Fat Bread Formulations Enriched With Lupin and Microalgae.” Lebensmittel‐Wissenschaft & Technologie 191: 115612.

[fsn370116-bib-0046] Pimentel, T. C. , W. K. A. Da Costa , C. E. Barão , M. Rosset , and M. Magnani . 2021. “Vegan Probiotic Products: A Modern Tendency or the Newest Challenge in Functional Foods.” Food Research International 140: 110033. 33648260 10.1016/j.foodres.2020.110033

[fsn370116-bib-0047] Ragusa, I. , G. N. Nardone , S. Zanatta , W. Bertin , and E. Amadio . 2021. “ *Spirulina* for Skin Care: A Bright Blue Future.” Cosmetics 8, no. 1: 7.

[fsn370116-bib-0048] Re, R. , N. Pellegrini , A. Proteggente , A. Pannala , M. Yang , and C. Rice‐Evans . 1999. “Antioxidant Activity Applying an Improved ABTS Radical Cation Decolorization Assay.” Free Radical Biology and Medicine 26, no. 9–10: 1231–1237. 10381194 10.1016/s0891-5849(98)00315-3

[fsn370116-bib-0049] Reboleira, J. , R. Freitas , S. Pinteus , et al. 2019. “ *Spirulina* .” In Nonvitamin and Nonmineral Nutritional Supplements, edited by S. M. Nabavi and A. S. Silva . Academic Press.

[fsn370116-bib-0050] Saharan, V. , and S. Jood . 2021. “Effect of Storage on *Spirulina platensis* Powder Supplemented Breads.” Journal of Food Science and Technology 58, no. 3: 978–984. 33678881 10.1007/s13197-020-04612-1 PMC7884492

[fsn370116-bib-0051] Sakkas, H. , P. Bozidis , C. Touzios , et al. 2020. “Nutritional Status and the Influence of the Vegan Diet on the Gut Microbiota and Human Health.” Medicina 56, no. 2: 88. 32098430 10.3390/medicina56020088 PMC7073751

[fsn370116-bib-0052] Saraç, M. G. , T. Dedebaş , E. Hastaoğlu , and E. Arslan . 2022. “Influence of Using Scarlet Runner Bean Flour on the Production and Physicochemical, Textural, and Sensorial Properties of Vegan Cakes: WASPAS‐SWARA Techniques.” International Journal of Gastronomy and Food Science 27: 100489.

[fsn370116-bib-0053] Sebastiani, G. , A. Herranz Barbero , C. Borrás‐Novell , et al. 2019. “The Effects of Vegetarian and Vegan Diet During Pregnancy on the Health of Mothers and Offspring.” Nutrients 11, no. 3: 557. 30845641 10.3390/nu11030557 PMC6470702

[fsn370116-bib-0054] Selmo, M. S. , and M. M. Salas‐Mellado . 2014. “Technological Quality of Bread From Rice Flour With *Spirulina* .” International Food Research Journal 21, no. 4: 1523–1528.

[fsn370116-bib-0055] Sengupta, S. , and J. Bhowal . 2017. “Optimization of Ingredient and Processing Parameters for the Production of *Spirulina platensis* Incorporated Soy Yogurt Using Response Surface Methodology.” Journal of Microbiology, Biotechnology and Food Sciences 6, no. 4: 1081.

[fsn370116-bib-0056] Sharma, P. , and N. Sharma . 2017. “Industrial and Biotechnological Applications of Algae: A Review.” Journal of Advanced Plant Biology 1, no. 1: 1.

[fsn370116-bib-0057] Silva, S. P. , A. F. do Valle , and D. Perrone . 2021. “Microencapsulated *Spirulina* Maxima Biomass as an Ingredient for the Production of Nutritionally Enriched and Sensorially Well‐Accepted Vegan Biscuits.” Lebensmittel‐Wissenschaft & Technologie 142: 110997.

[fsn370116-bib-0058] Singleton, V. L. , and J. A. Rossi . 1965. “Colorimetry of Total Phenolics With Phosphomolybdic‐Phosphotungstic Acid Reagents.” American Journal of Enology and Viticulture 16, no. 3: 144–158.

[fsn370116-bib-0059] Soni, R. A. , K. Sudhakar , and R. S. Rana . 2017. “ *Spirulina*—From Growth to Nutritional Product: A Review.” Trends in Food Science & Technology 69: 157–171.

[fsn370116-bib-0060] Su, K. , Z. Fan , M. Usman , et al. 2025. “Effect of *Spirulina platensis* on the Structure and Aggregation of Gluten Proteins to Improve Texture and Physiochemical Properties of Wheat Noodles.” Food Hydrocolloids 162: 110959. 10.1016/j.foodhyd.2024.110959.

[fsn370116-bib-0061] Tork, M. B. , M. Vazifedoost , M. A. Hesarinejad , Z. Didar , and M. S. Zenoozian . 2022. “Fabrication of Dragee Containing *Spirulina platensis* Microalgae to Enrich Corn Snack and Evaluate Its Sensorial, Physicochemical, and Nutritional Properties.” Food 11, no. 13: 1909. 10.3390/foods11131909. PMC9265436 35804726

[fsn370116-bib-0062] Trotta, T. , C. Porro , A. Cianciulli , and M. A. Panaro . 2022. “Beneficial Effects of *Spirulina* Consumption on Brain Health.” Nutrients 14, no. 3: 676. 35277035 10.3390/nu14030676 PMC8839264

[fsn370116-bib-0063] Winarni Agustini, T. , W. Farid Ma'ruf , W. Widayat , M. Suzery , H. Hadiyanto , and S. Benjakul . 2016. “Application of *Spirulina platensis* on Ice Cream and Soft Cheese With Respect to Their Nutritional and Sensory Perspectives.” Jurnal Teknologi 78, no. 4–2: 245–251.

[fsn370116-bib-0064] Yazici, G. N. , and M. S. Ozer . 2021. “A Review of Egg Replacement in Cake Production: Effects on Batter and Cake Properties.” Trends in Food Science & Technology, 111: 346–359.

[fsn370116-bib-0067] Zlateva, D ., P. Ivanova , R. Chochkov , and D. Stefanova , 2020. “Effect of Spirulina platensis and kelp on the antioxidant activity of wheat bread.” Ukrainian Food Journal 9, no.3: 636–650.

