# Supplementary material for: Optimization of Spirulina‐Enriched Vegan Cake Formulation Using Response Surface Methodology
Source: Food Sci Nutr. 2025 Mar 24;13(4):e70116. doi: 10.1002/fsn3.70116 (PMC11932053; doi:10.1002/fsn3.70116)
Supplement: Supplementary file 1 — Data S1. [file FSN3-13-e70116-s001.docx]

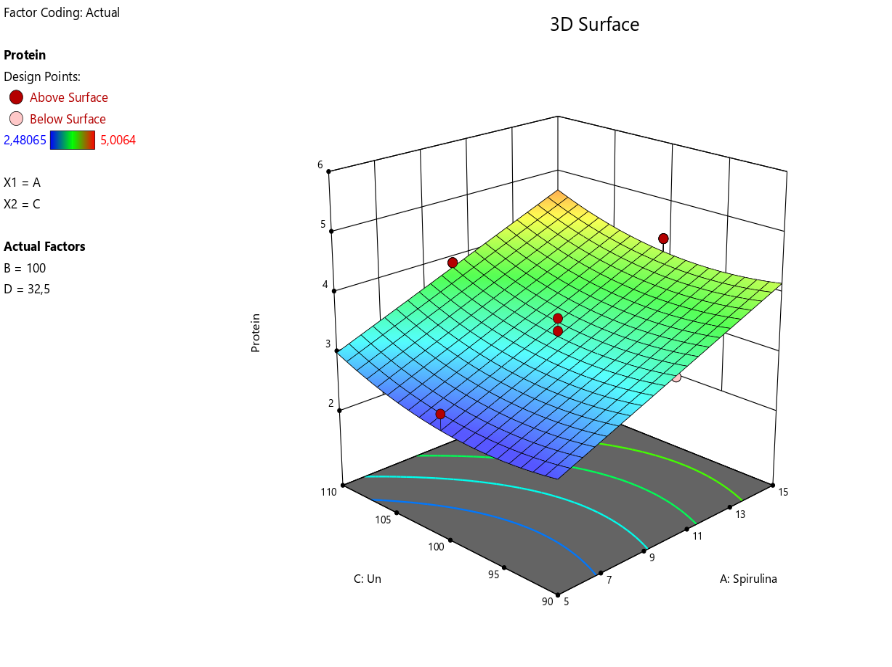

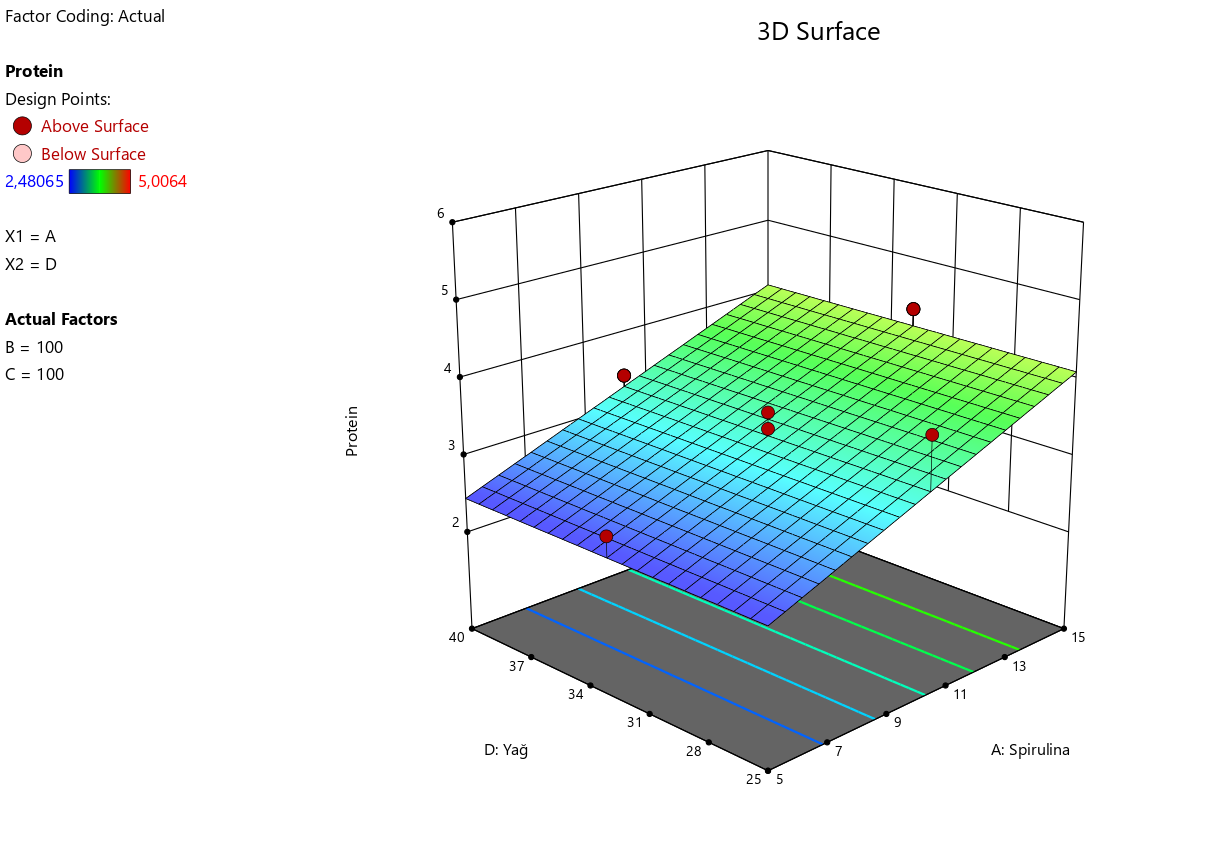

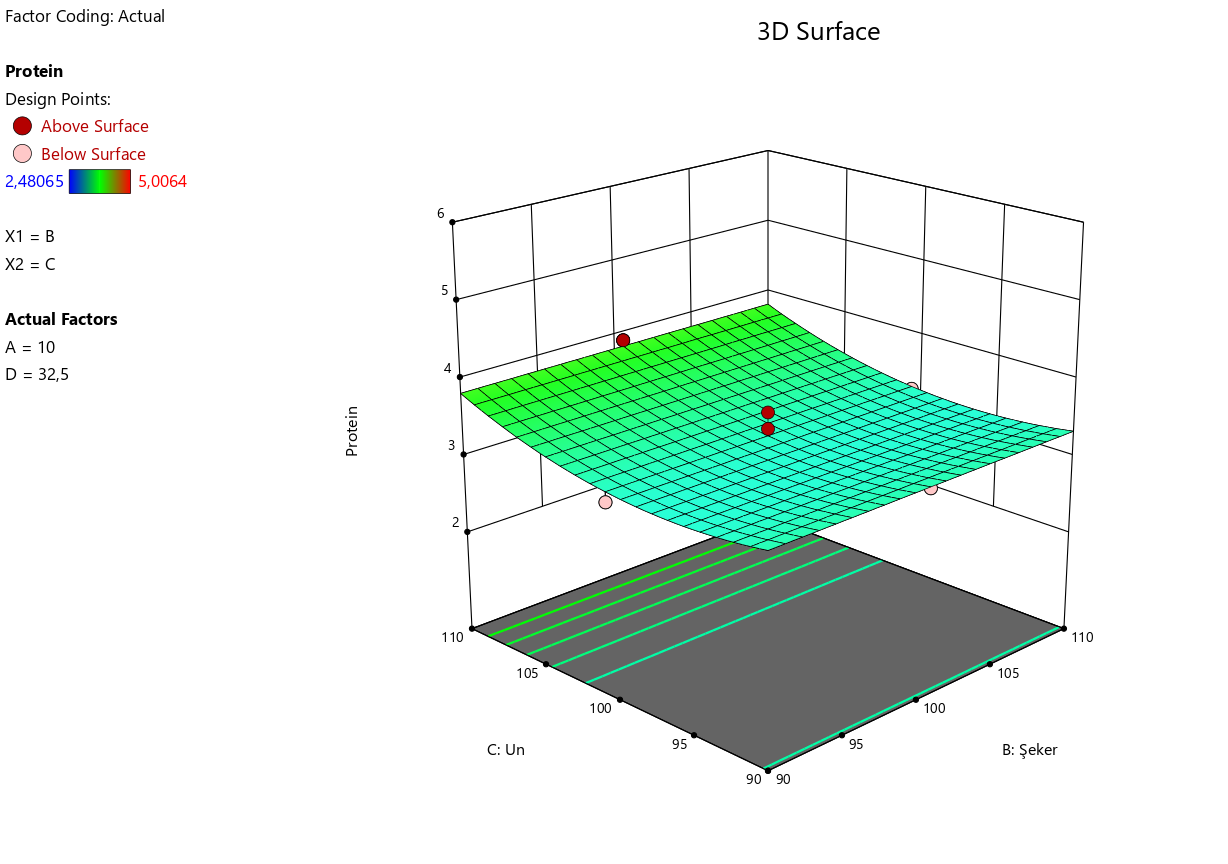

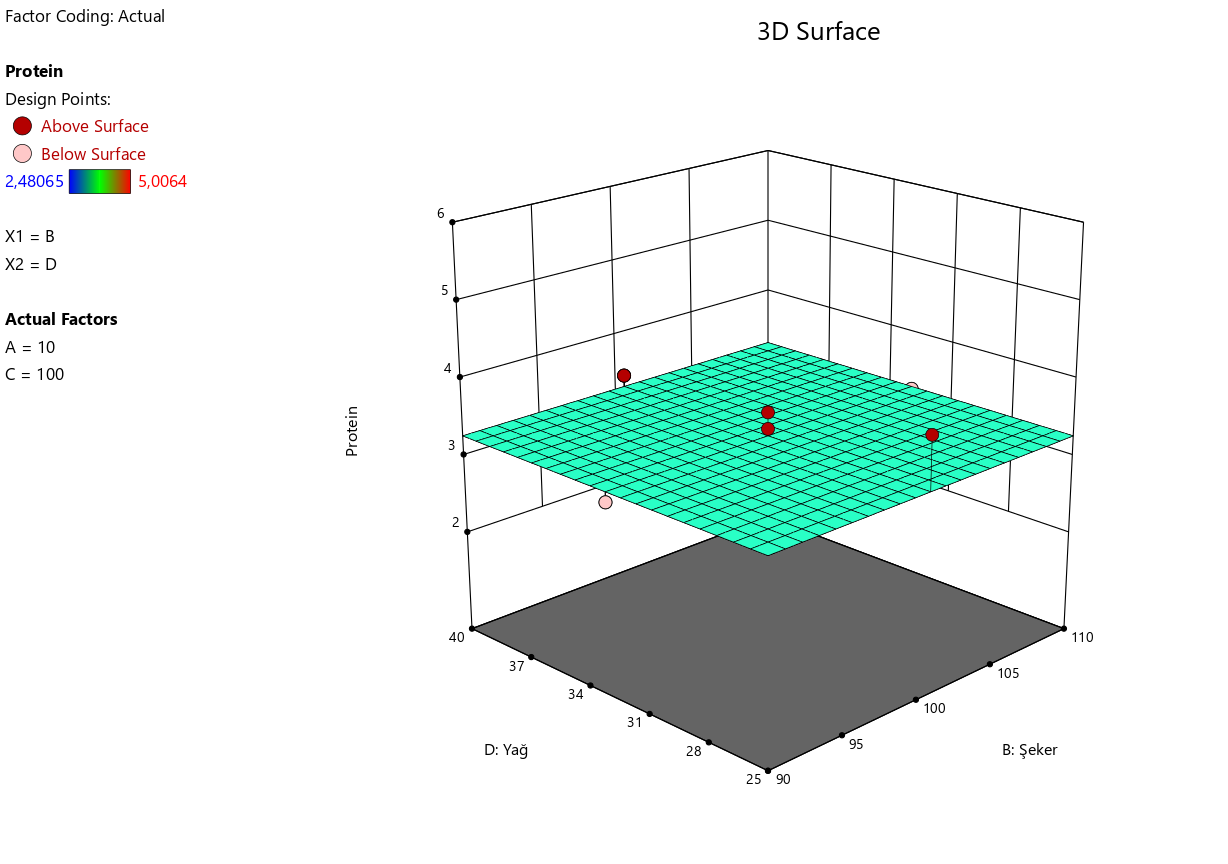

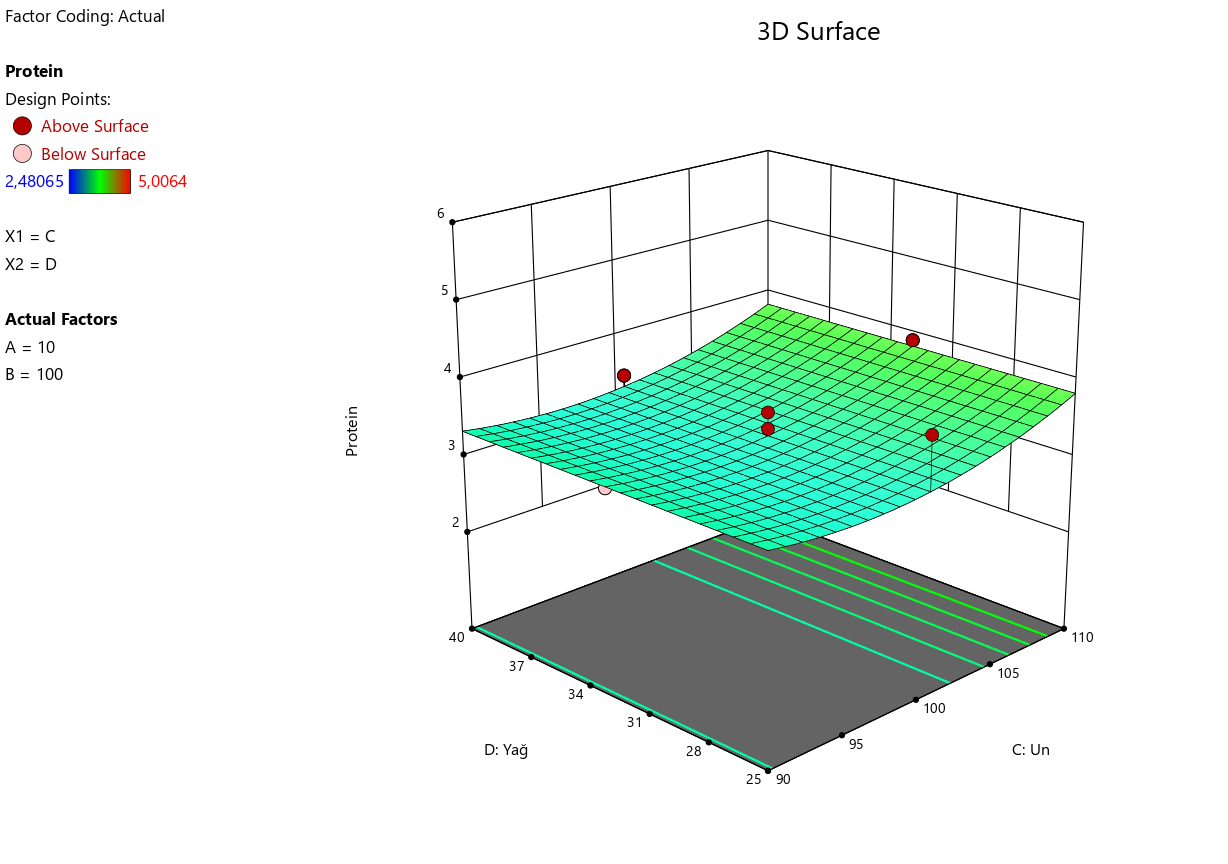


Protein

C: Flour

A: Spirulina

Protein

A: Spirulina

D: Oil

Protein

B: Sugar

C: Flour

Protein

B: Sugar

D: Oil

Protein

C: Flour

D: Oil


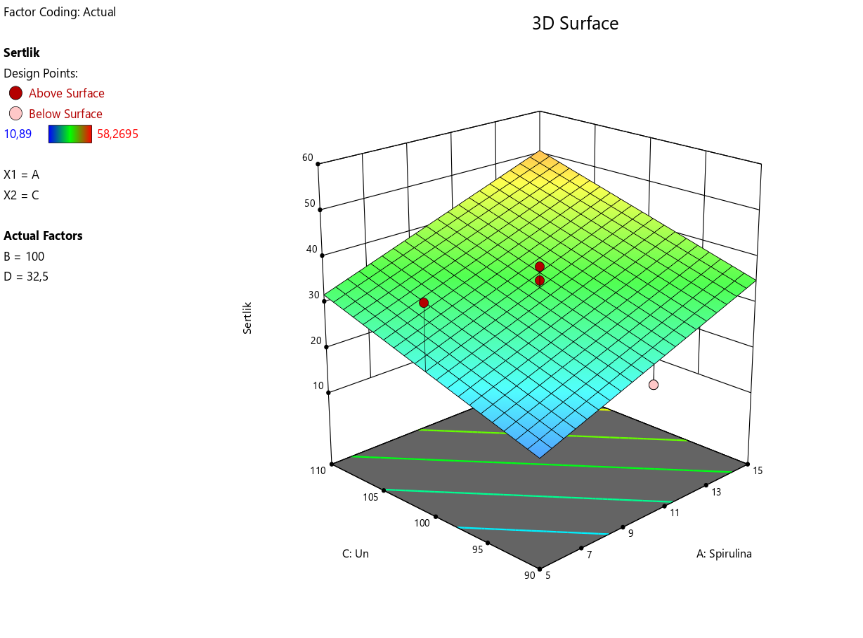

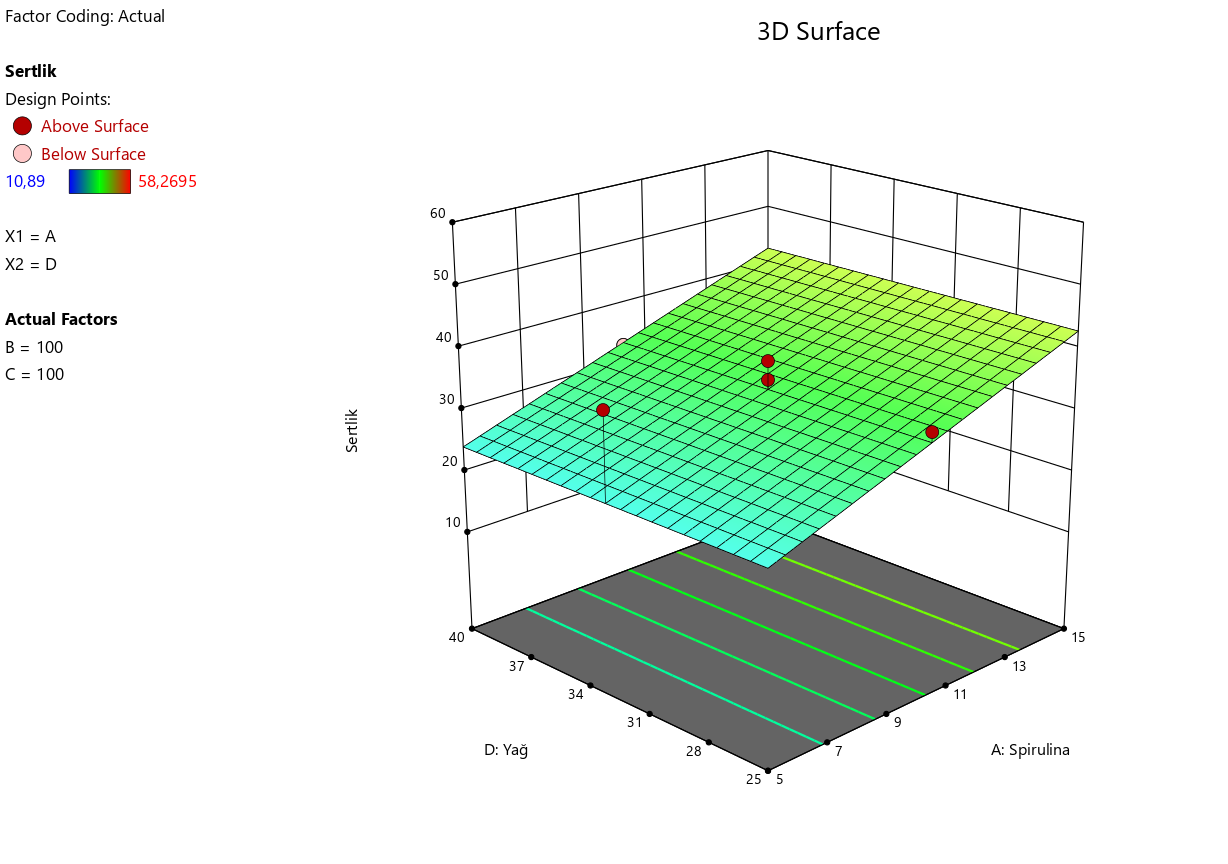

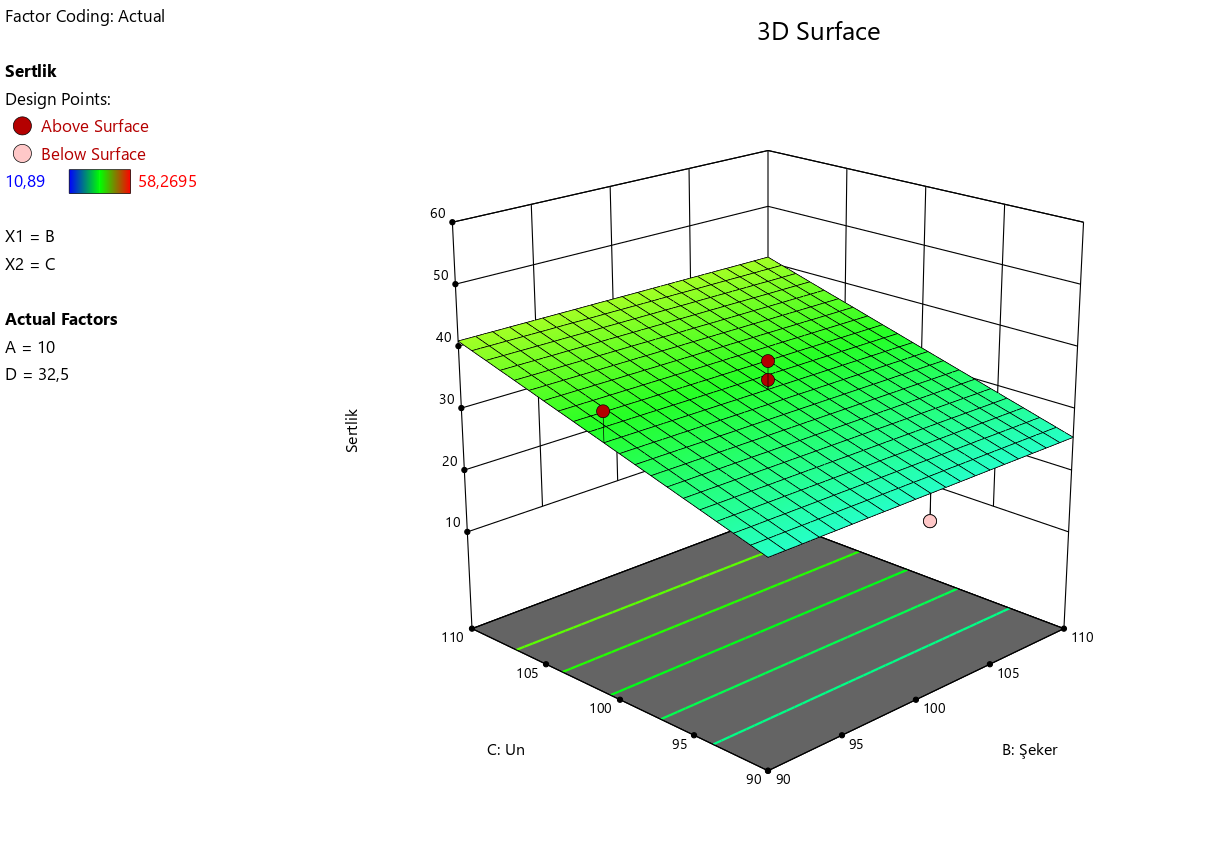

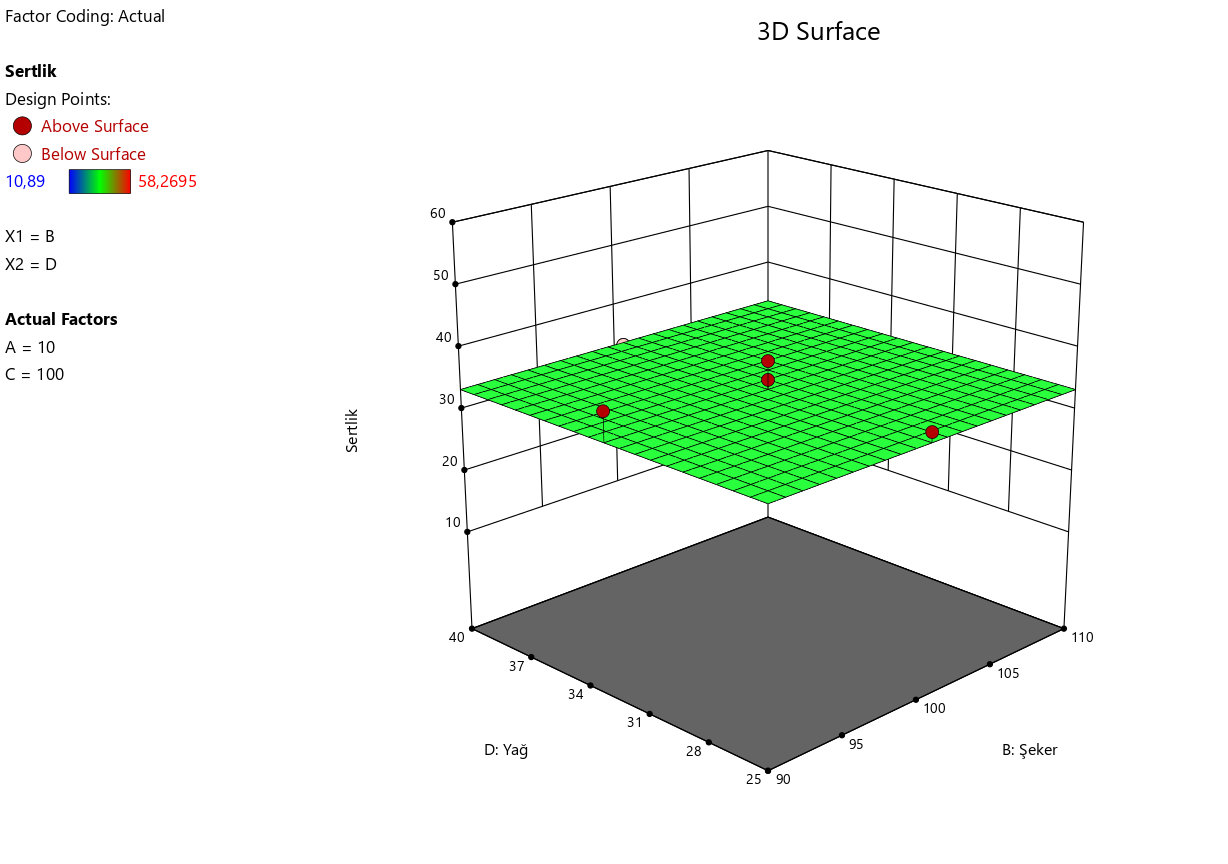

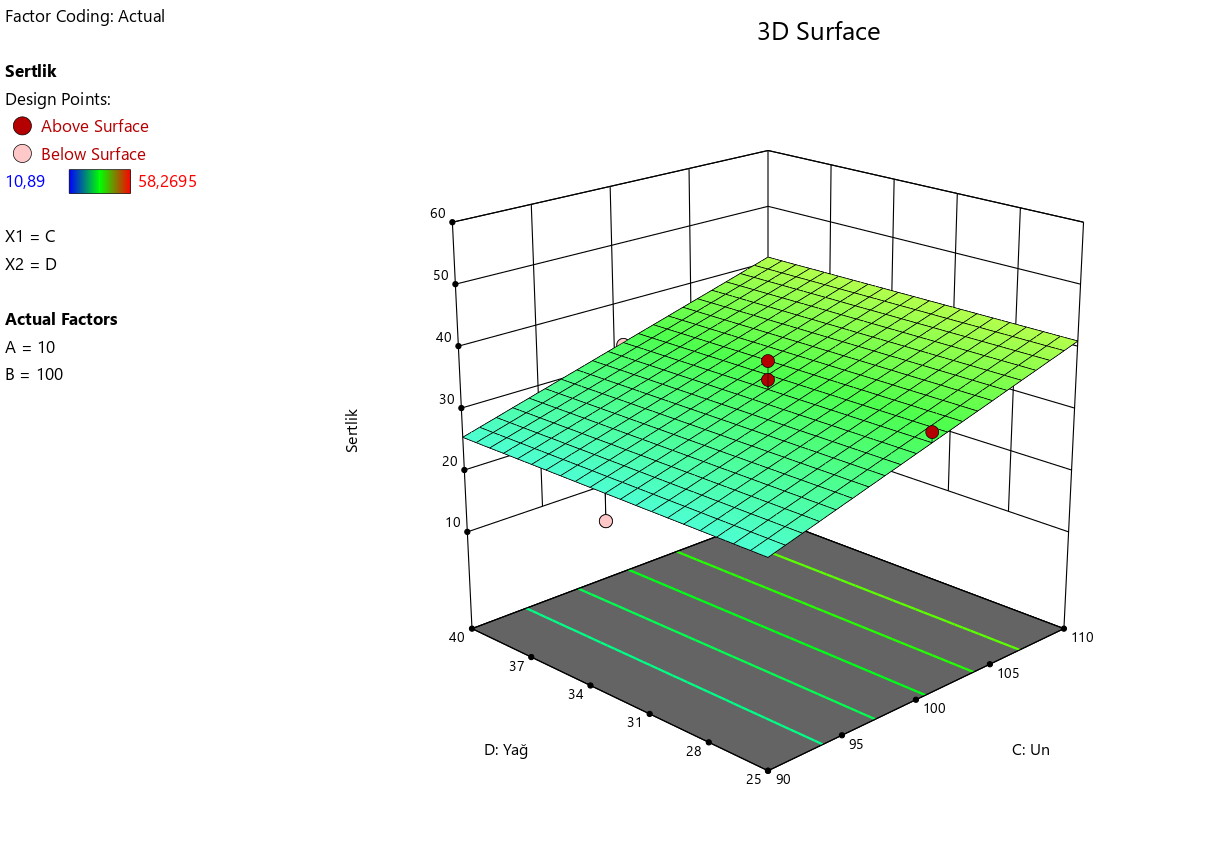


Hardness

C: Flour

D: Oil

Hardness

B: Sugar

D: Oil

Hardness

A: Spirulina

D: Oil

Hardness

B: Sugar

C: Flour

Hardness

A: Spirulina

C: Flour


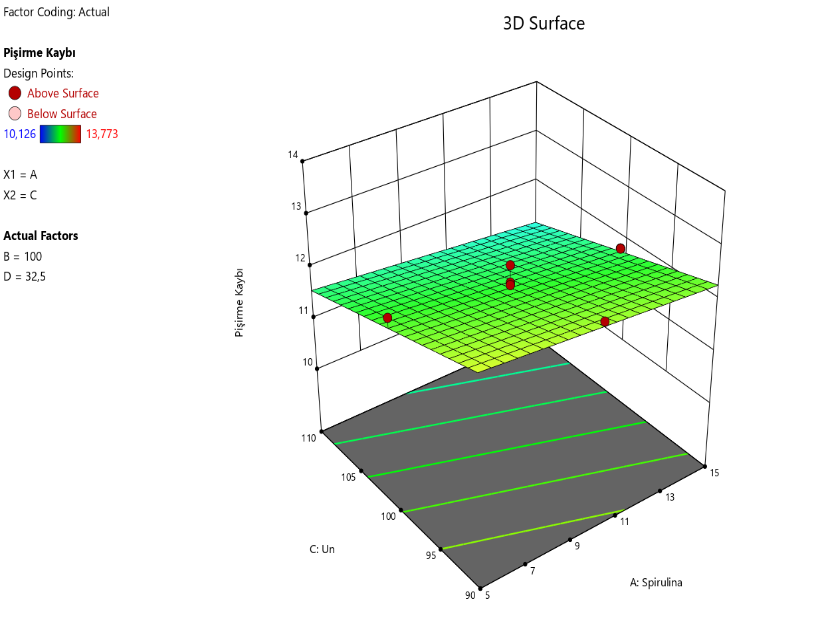

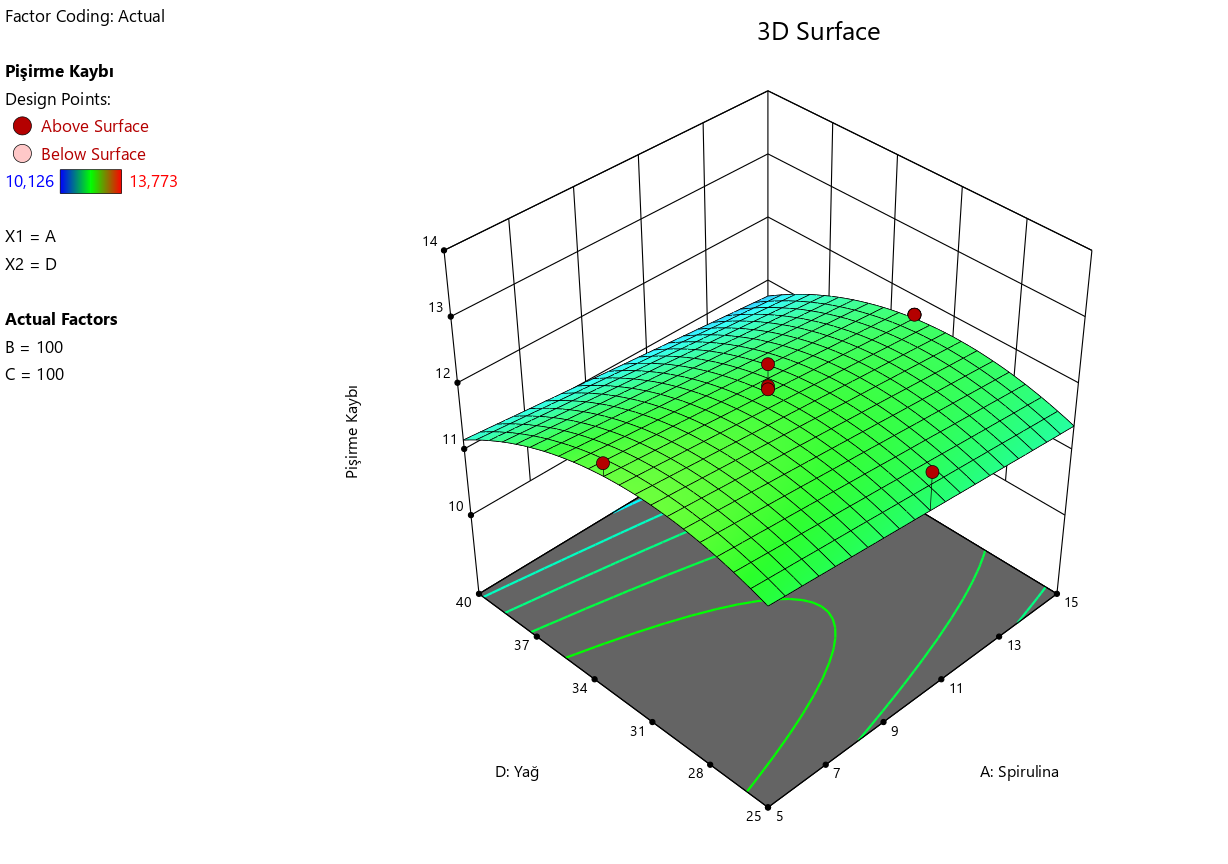

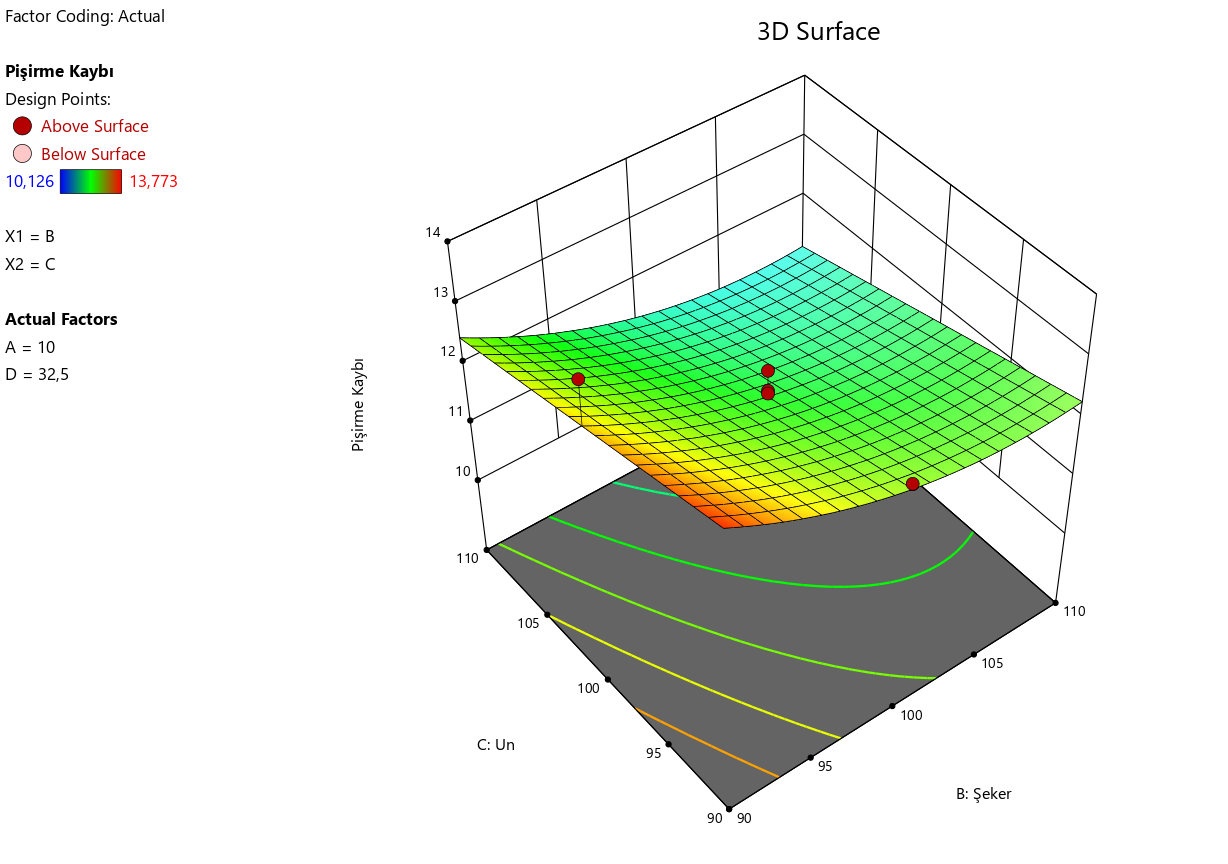

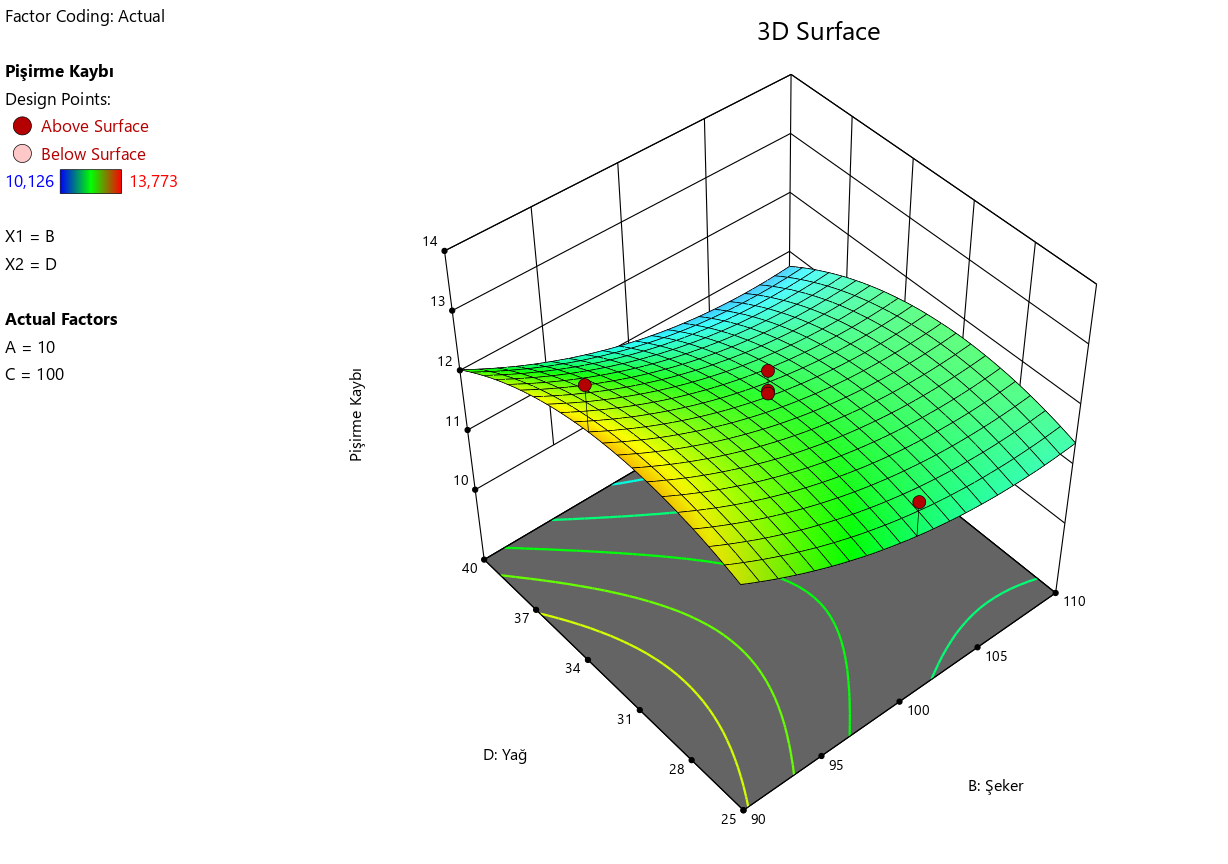

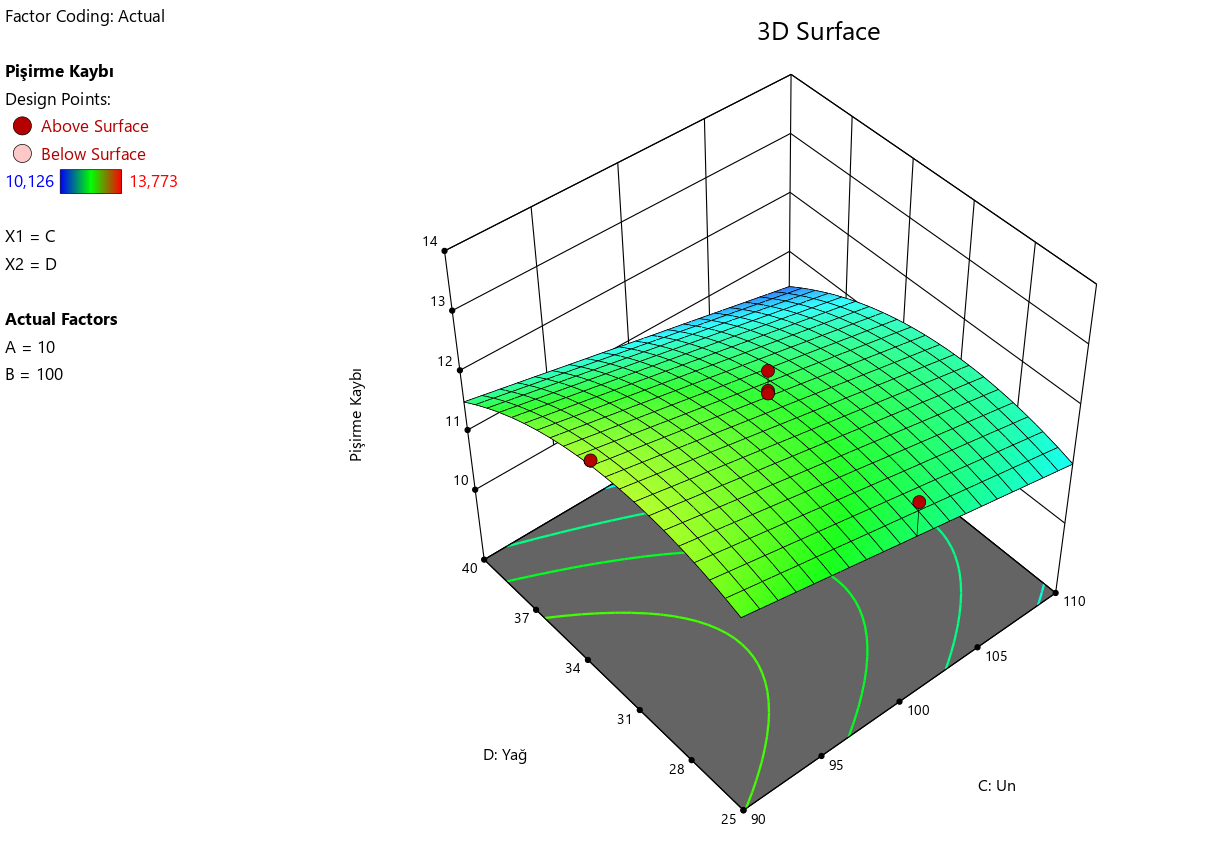


Baking Loss (%)

D: Oil

C: Flour

Baking Loss (%)

A: Spirulina

C: Flour

Baking Loss (%)

A: Spirulina

D: Oil

Baking Loss (%)

B: Sugar

C: Flour

Baking Loss (%)

B: Sugar

D: Oil


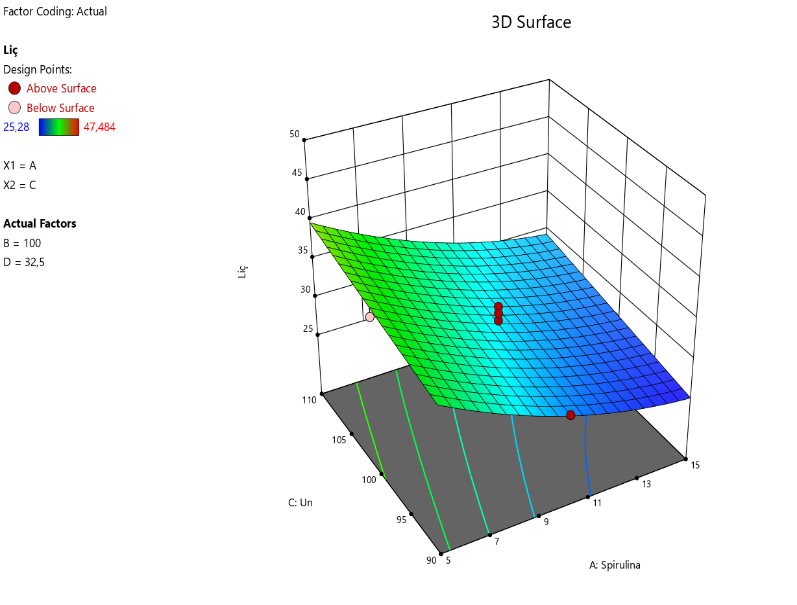

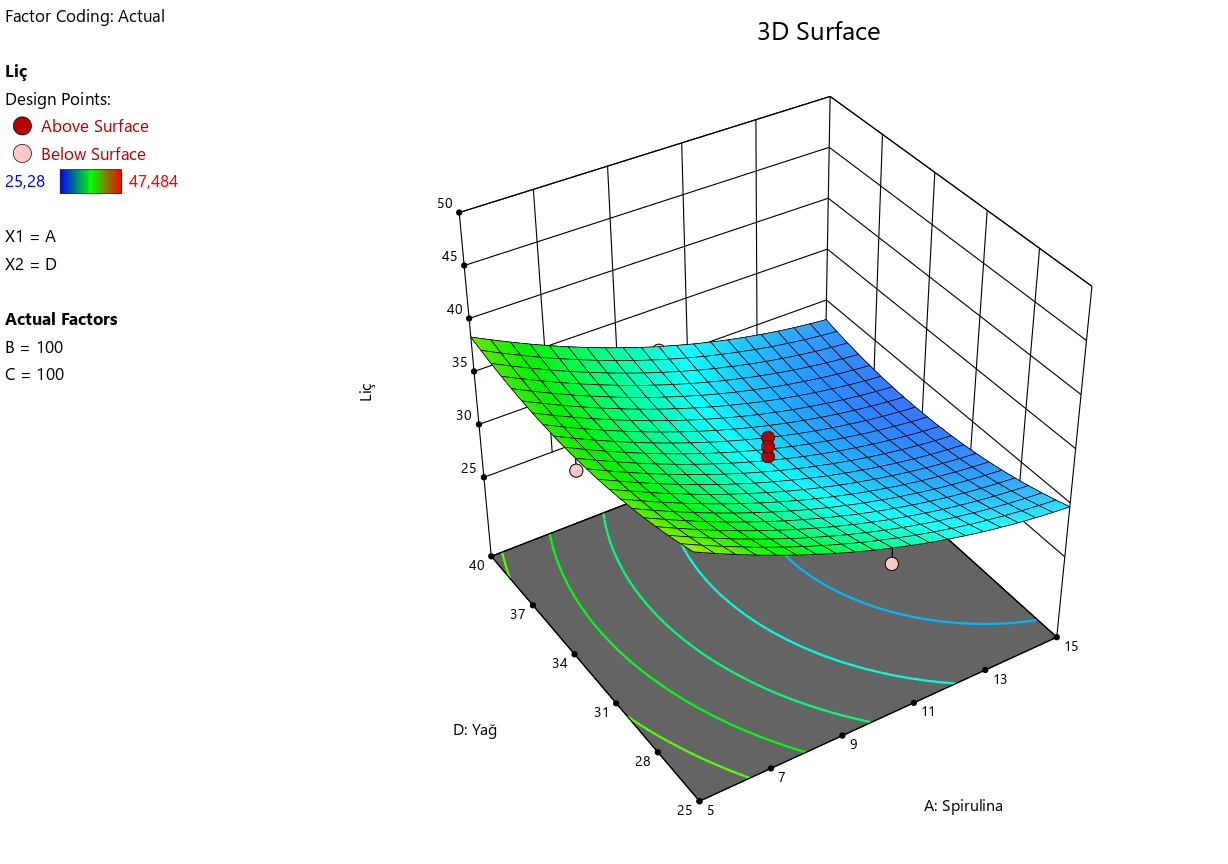

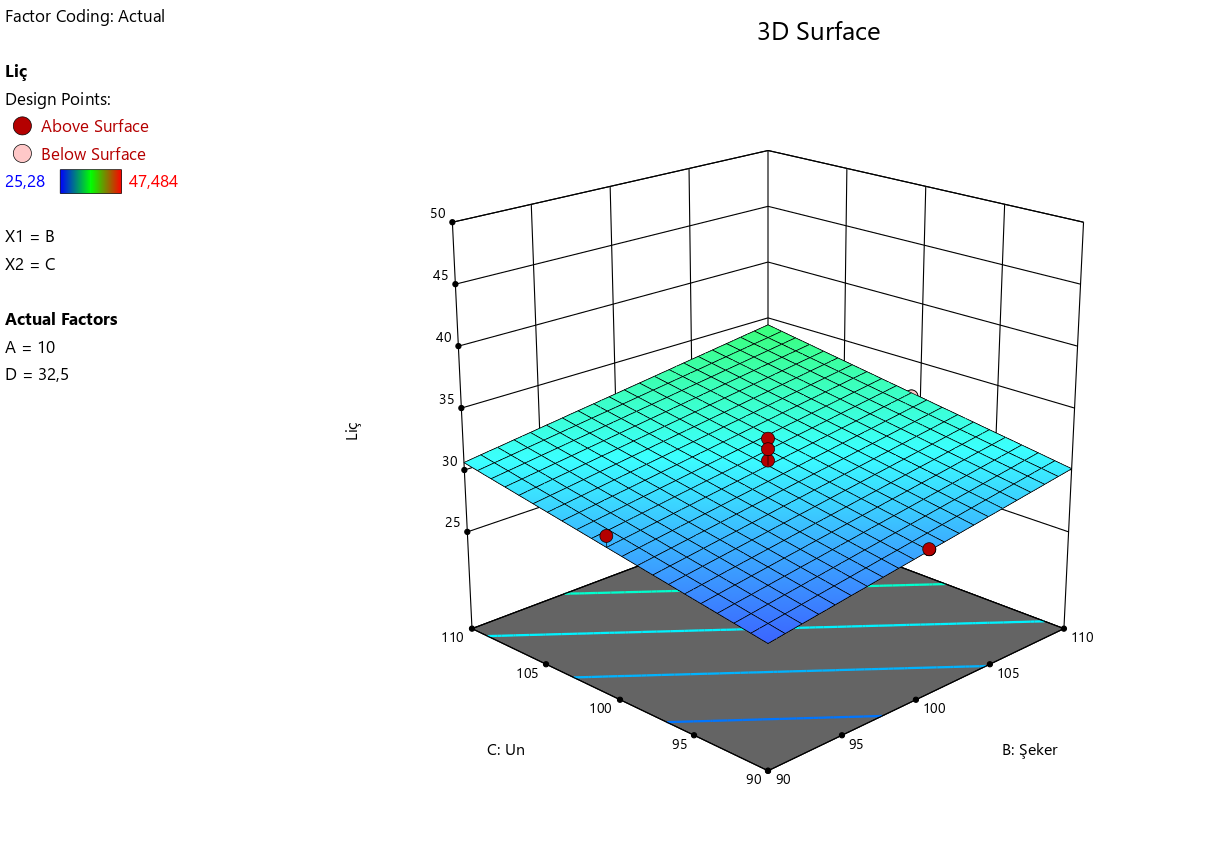

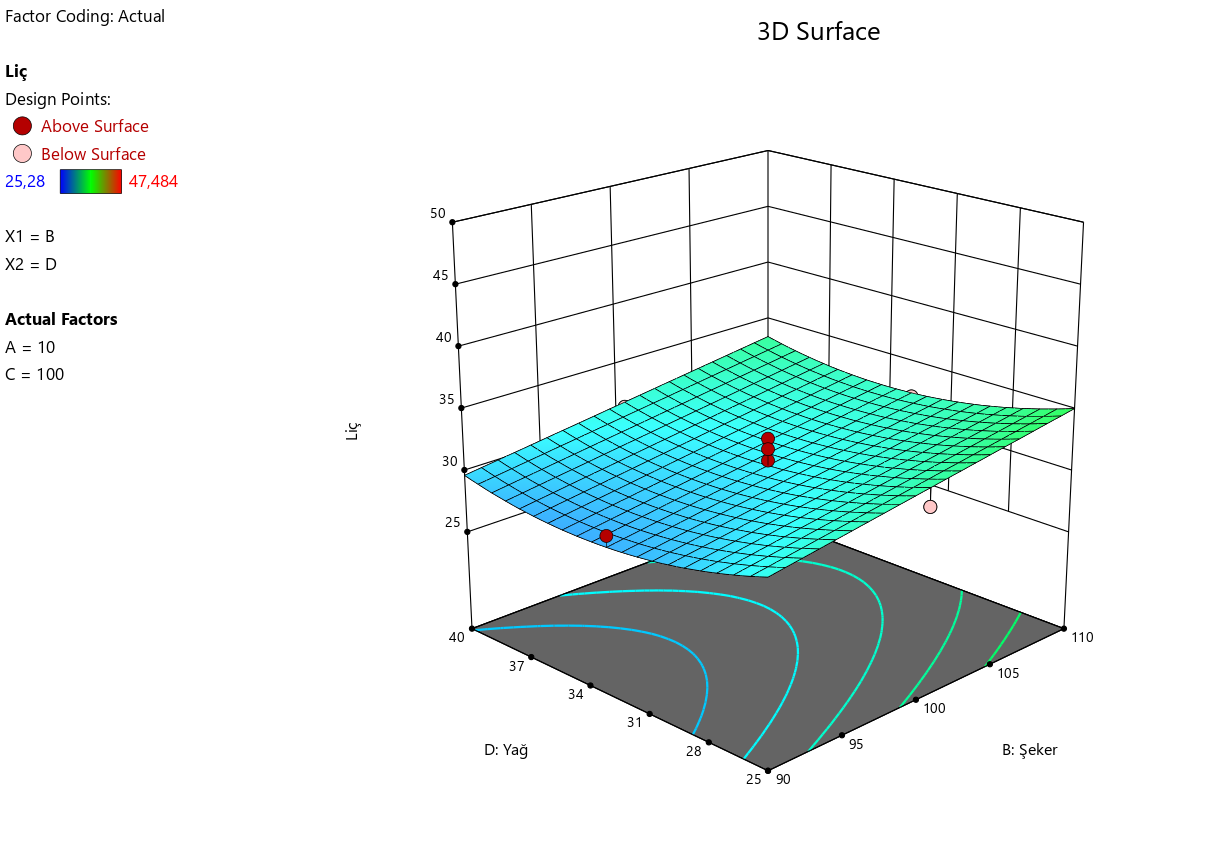

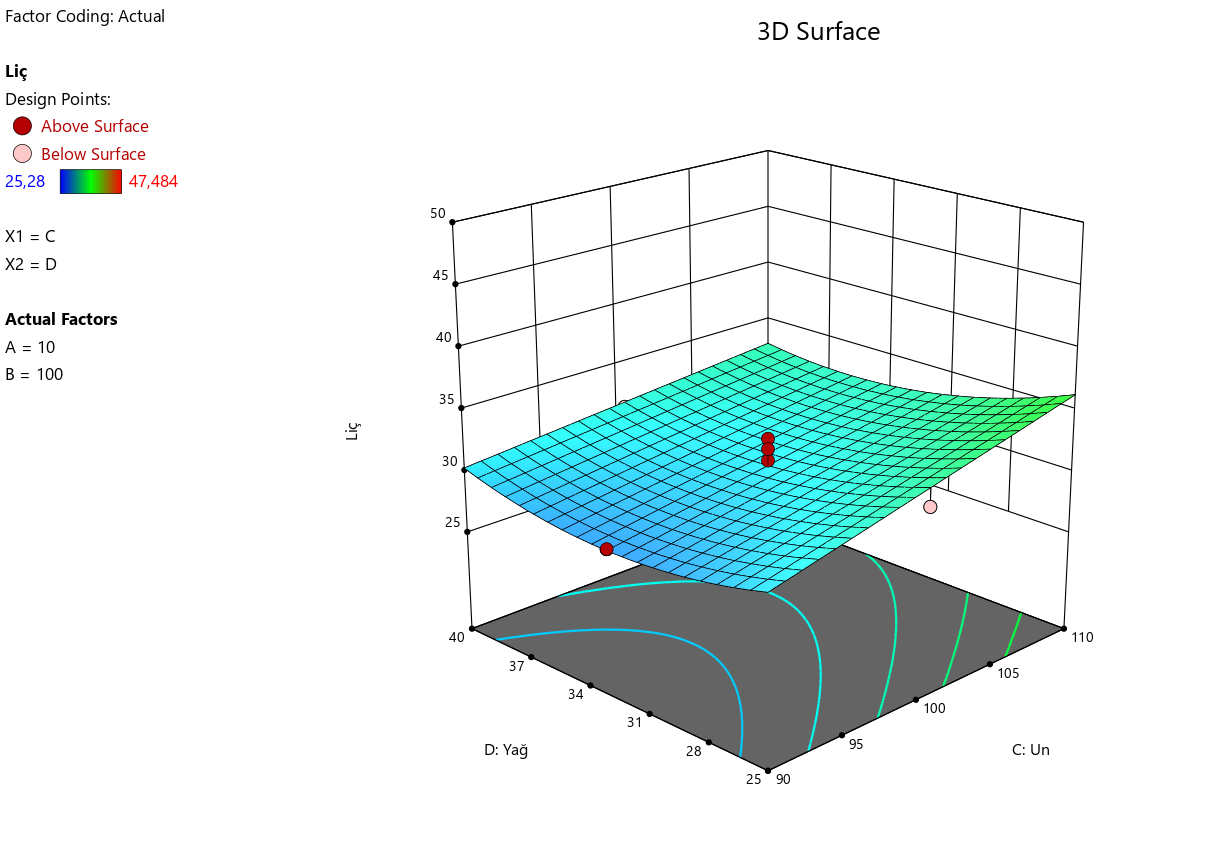


Lcrumb*

A: Spirulina

C: Flour

D: Oil

Lcrumb*

A: Spirulina

B: Sugar

Lcrumb*

C: Flour

Lcrumb*

D: Oil

C: Flour

Lcrumb*

B: Sugar

D: Oil


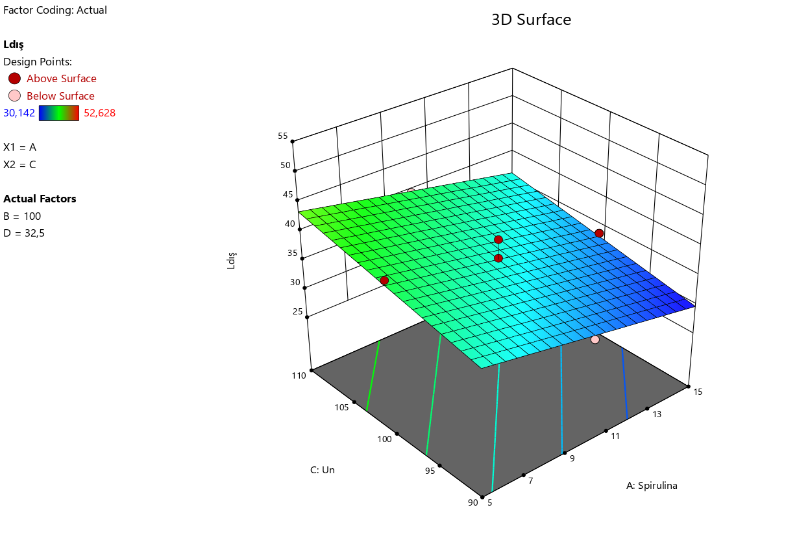

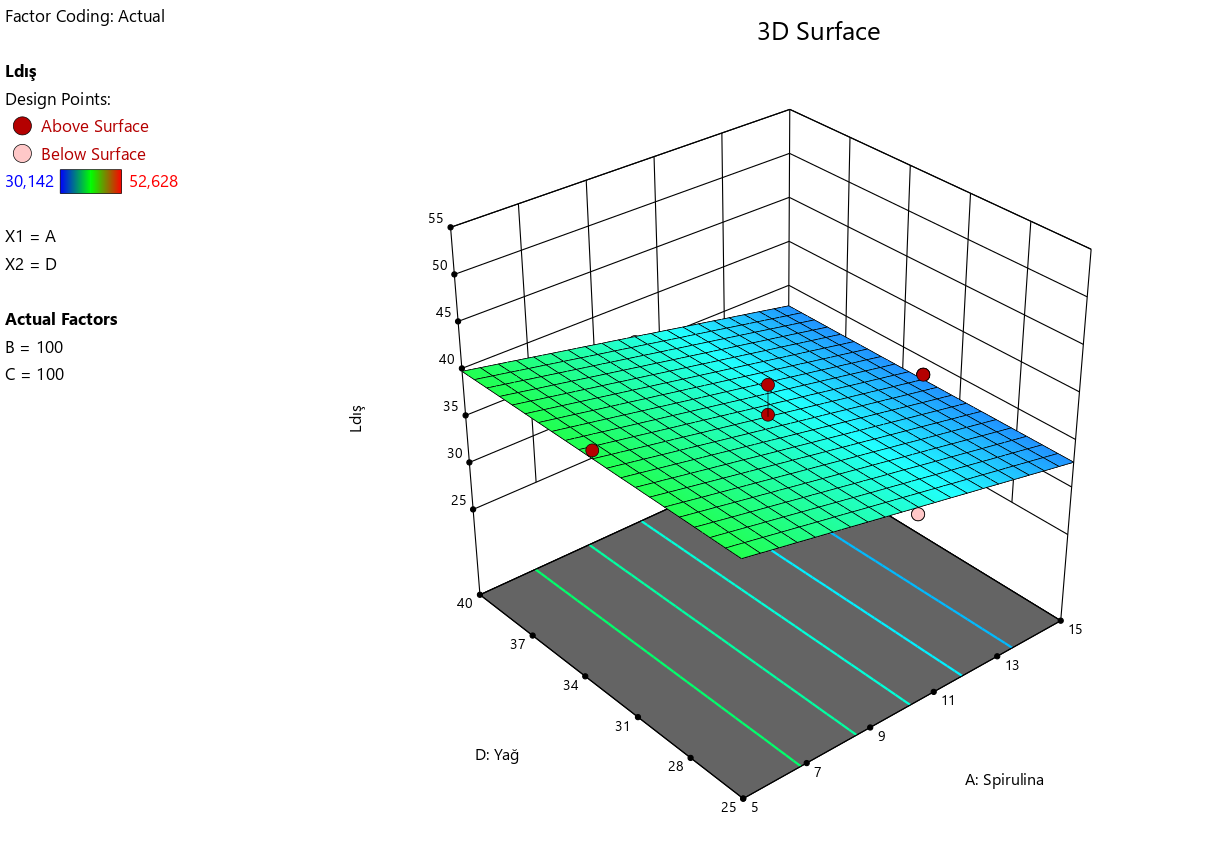

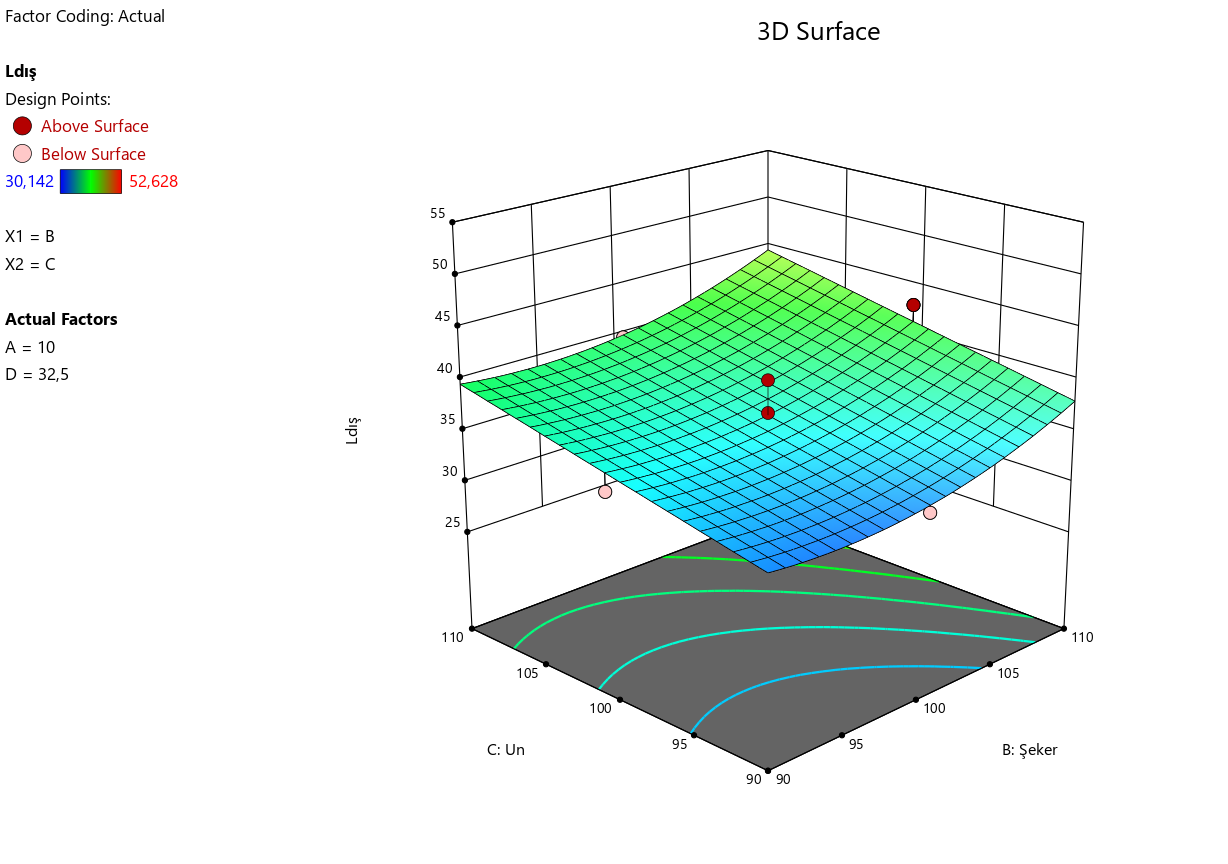

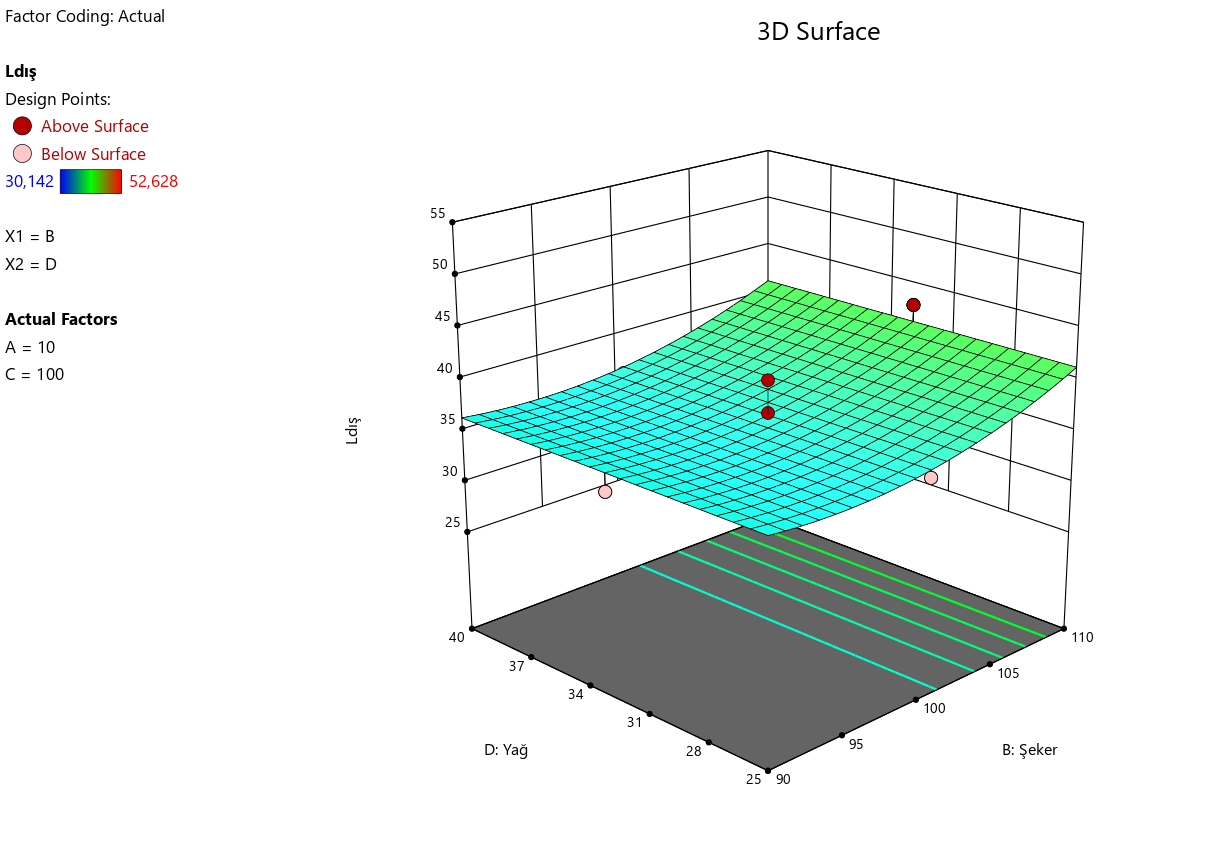

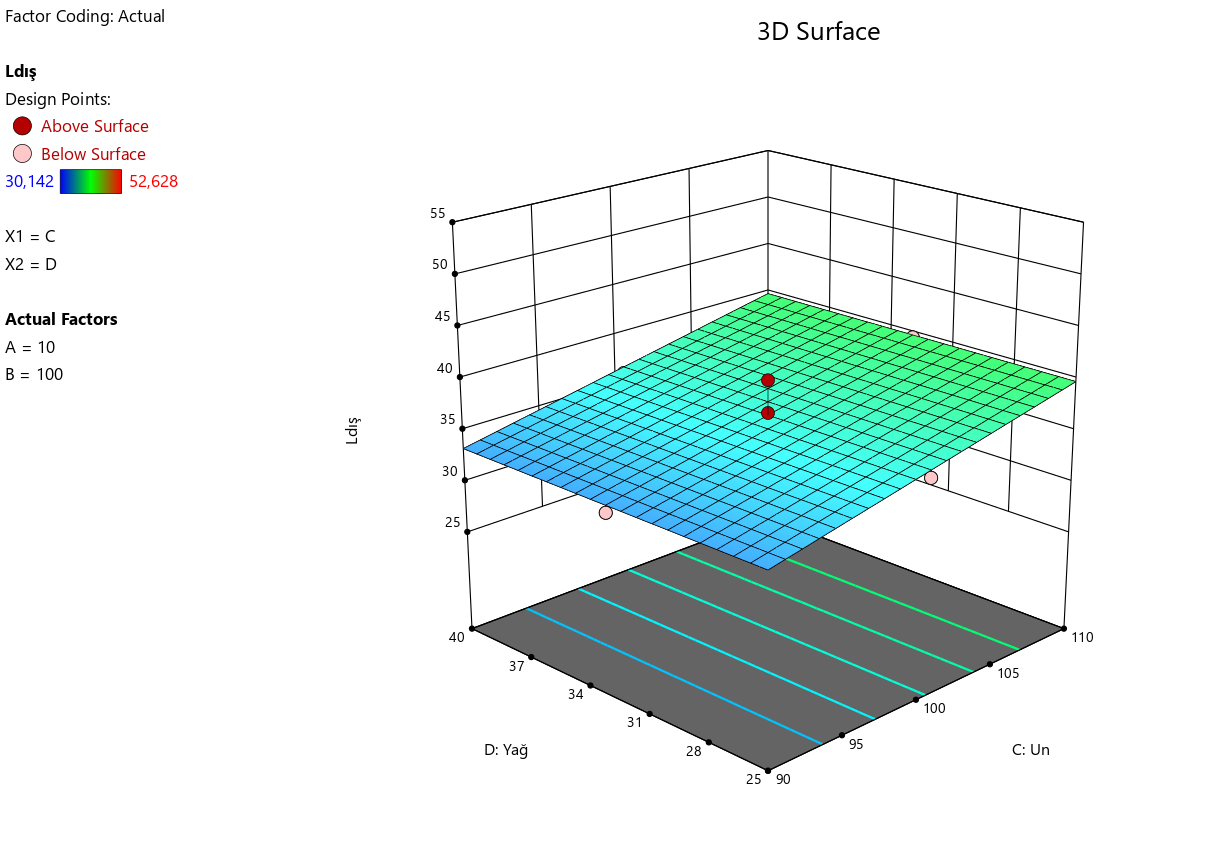


A: Spirulina

D: Oil

C: Flour

Lcrust*

Lcrust*

Lcrust*

Lcrust*

Lcrust*

A: Spirulina

B: Sugar

B: Sugar

C: Flour

C: Flour

D: Oil

D: Oil


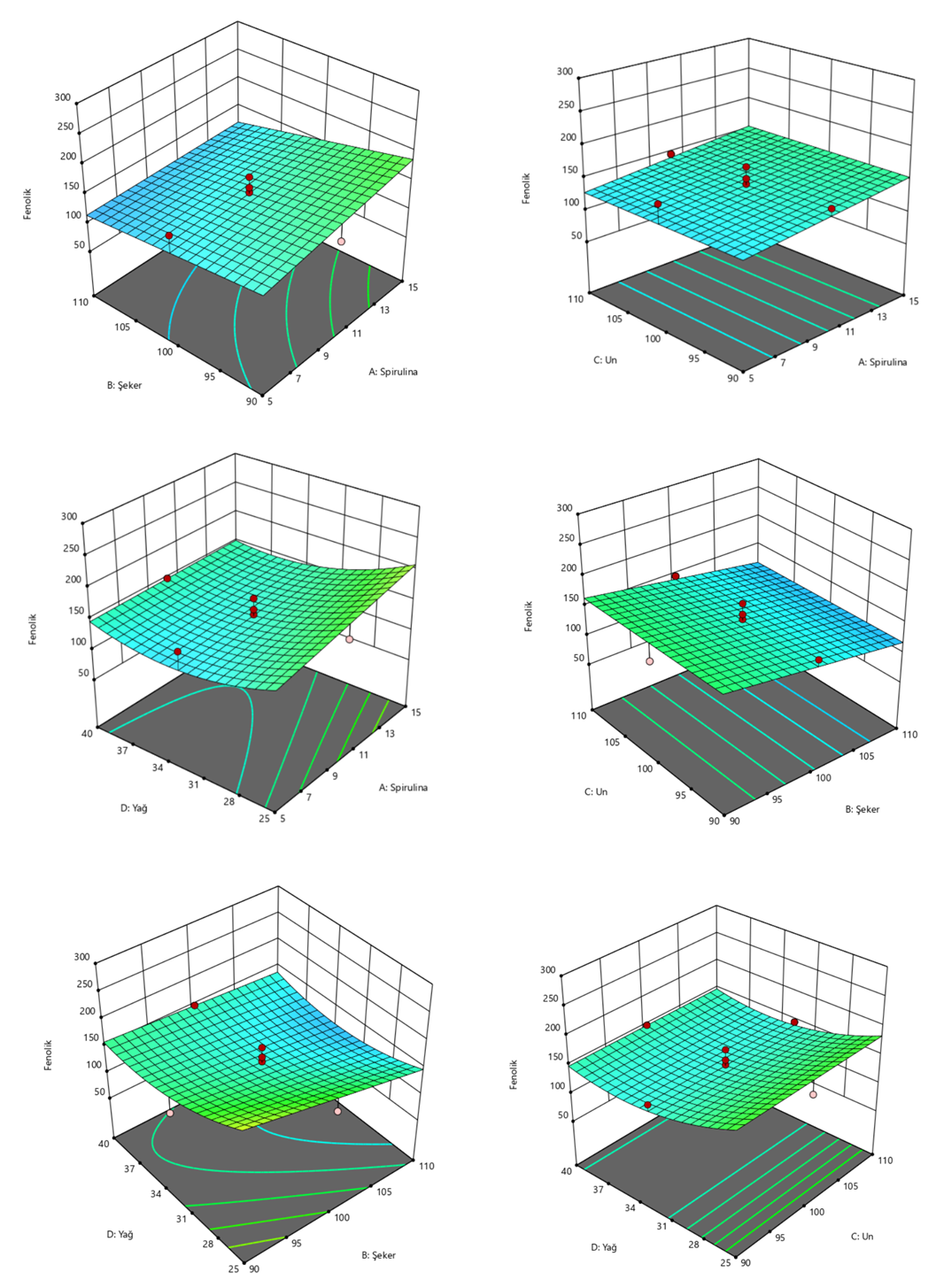


B: Sugar

Total phenolic content

D: Oil

C: Flour

A: Spirulina

Total phenolic content

Total phenolic content

Total phenolic content

Total phenolic content

Total phenolic content

D: Oil

D: Oil

B: Sugar

C: Flour

A: Spirulina

A: Spirulina

B: Sugar

C: Flour


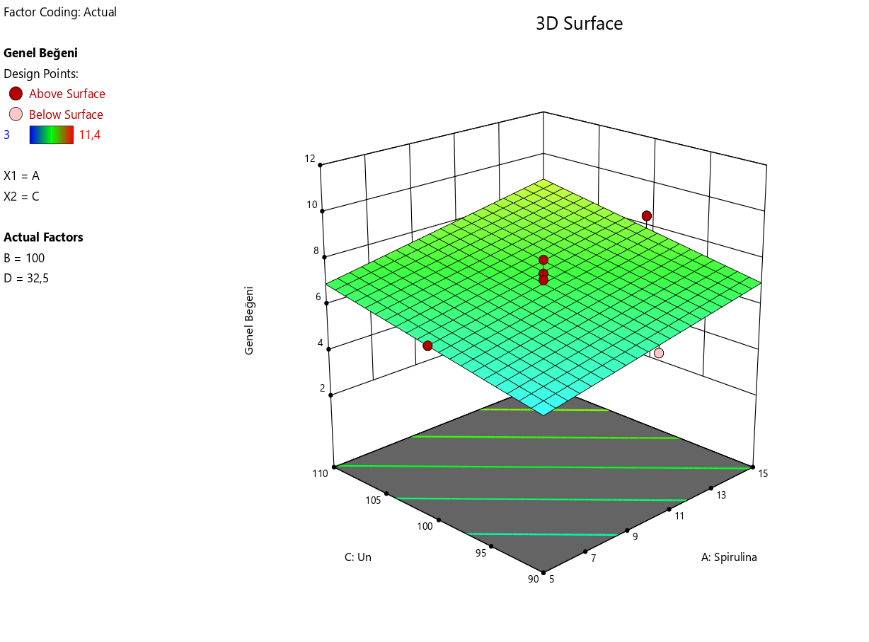

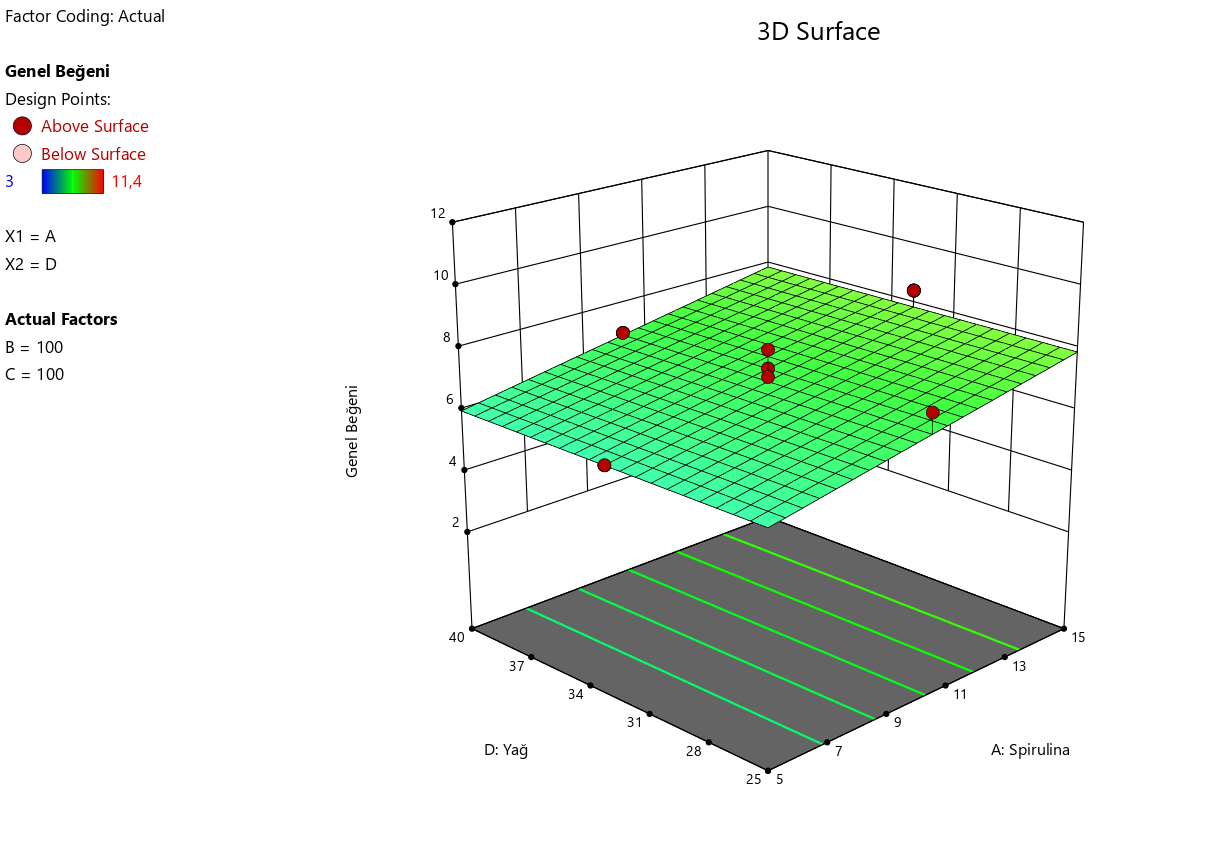

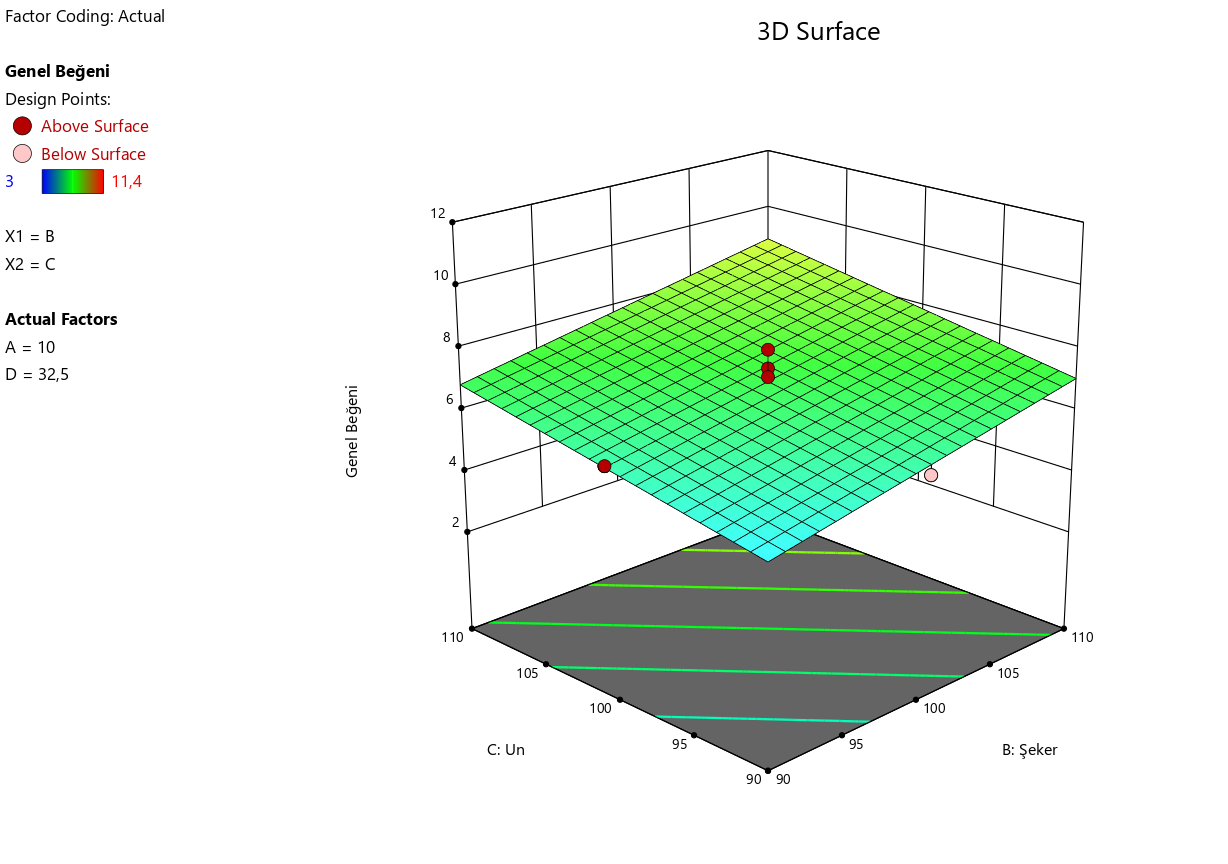

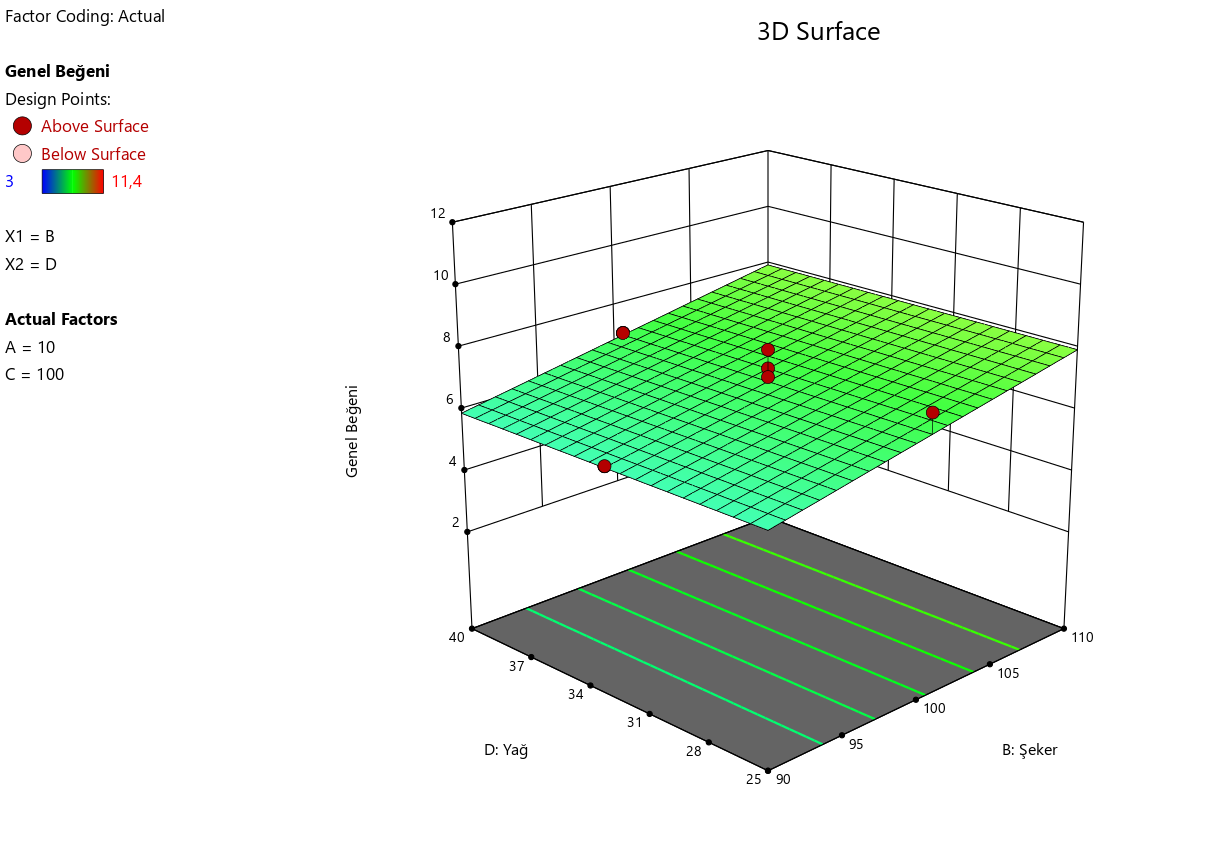

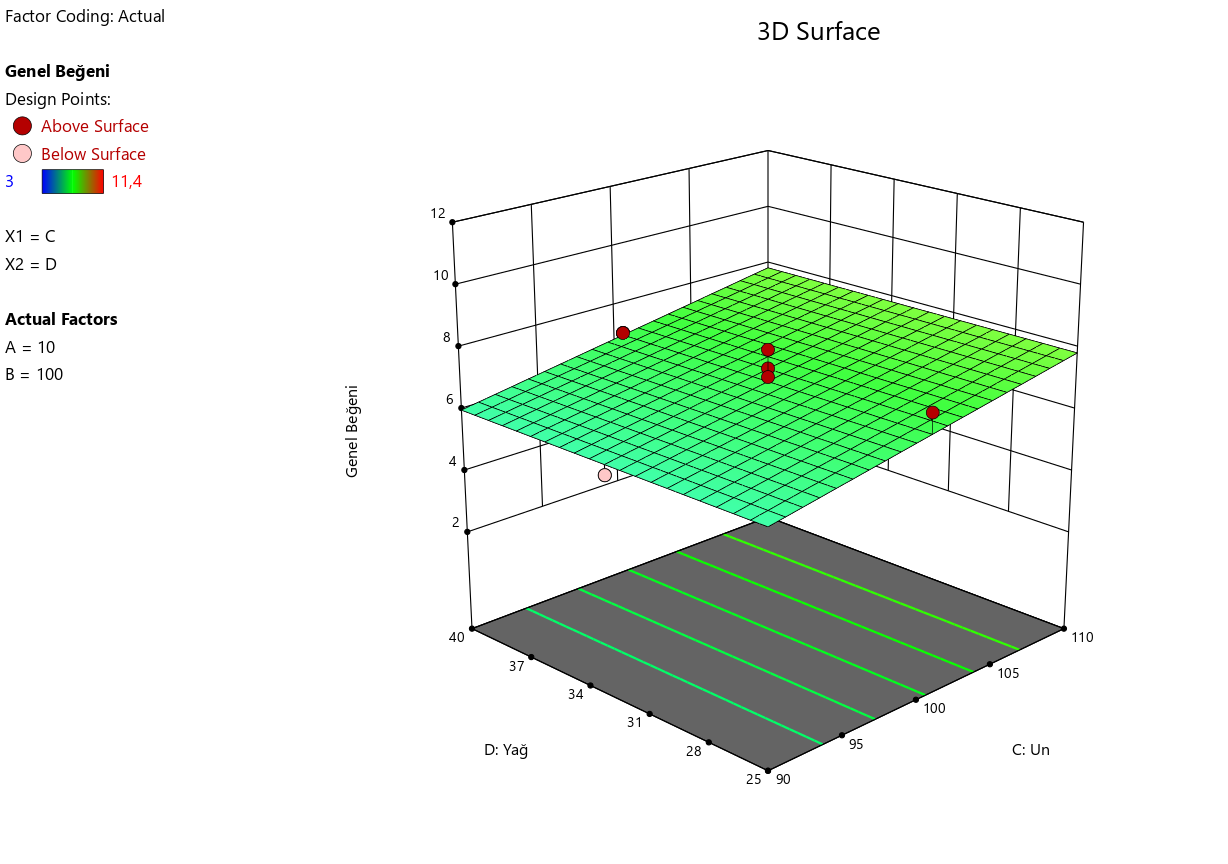


Overall Acceptability

D: Oil

C: Flour

Overall Acceptability

Overall Acceptability

Overall Acceptability

Overall Acceptability

A: Spirulina

B: Sugar

A: Spirulina

C: Flour

C: Flour

D: Oil

D: Oil

B: Sugar


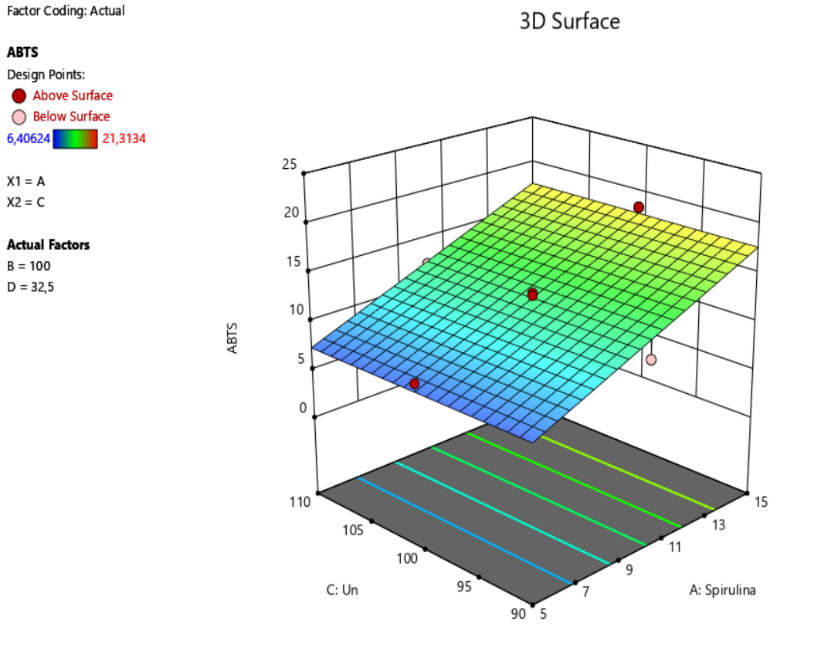

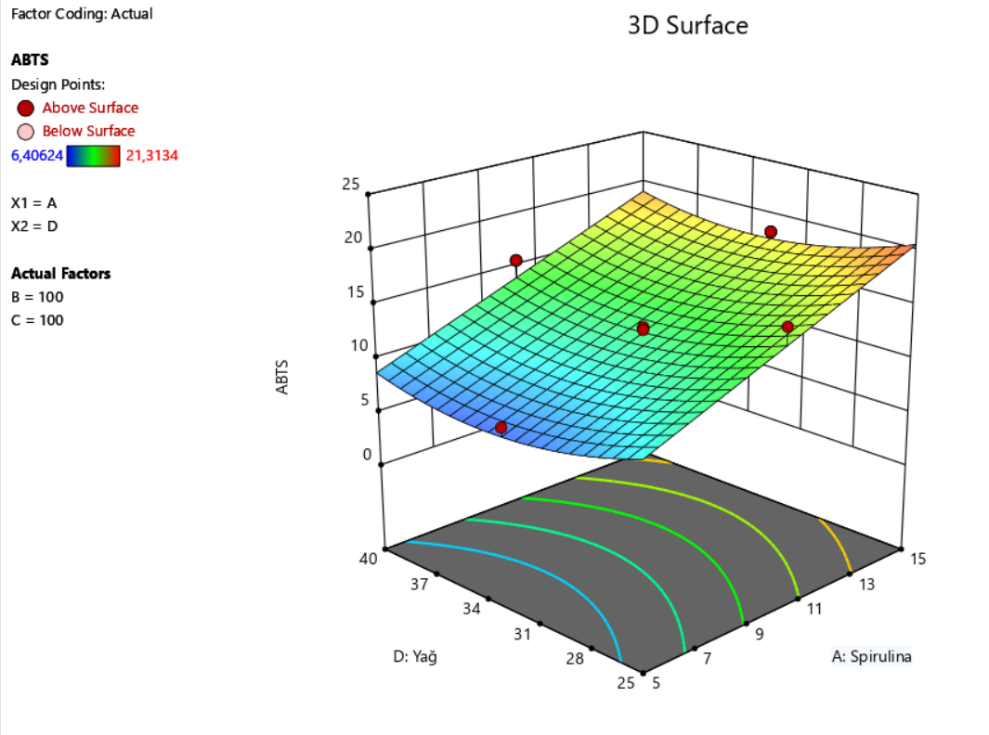

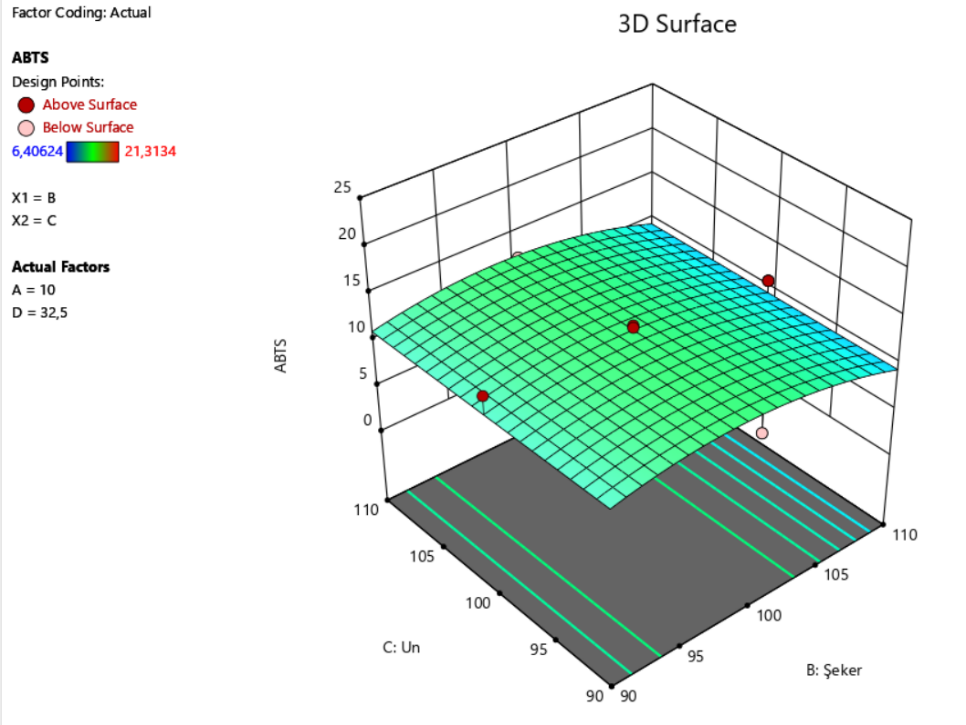

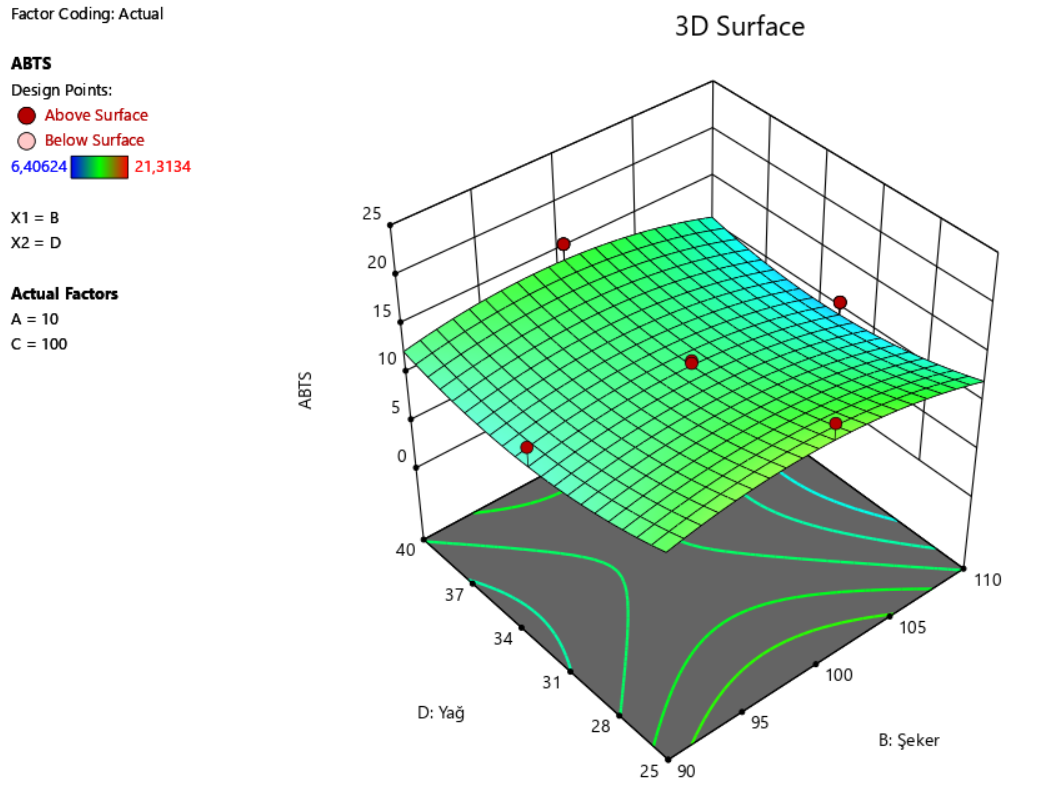

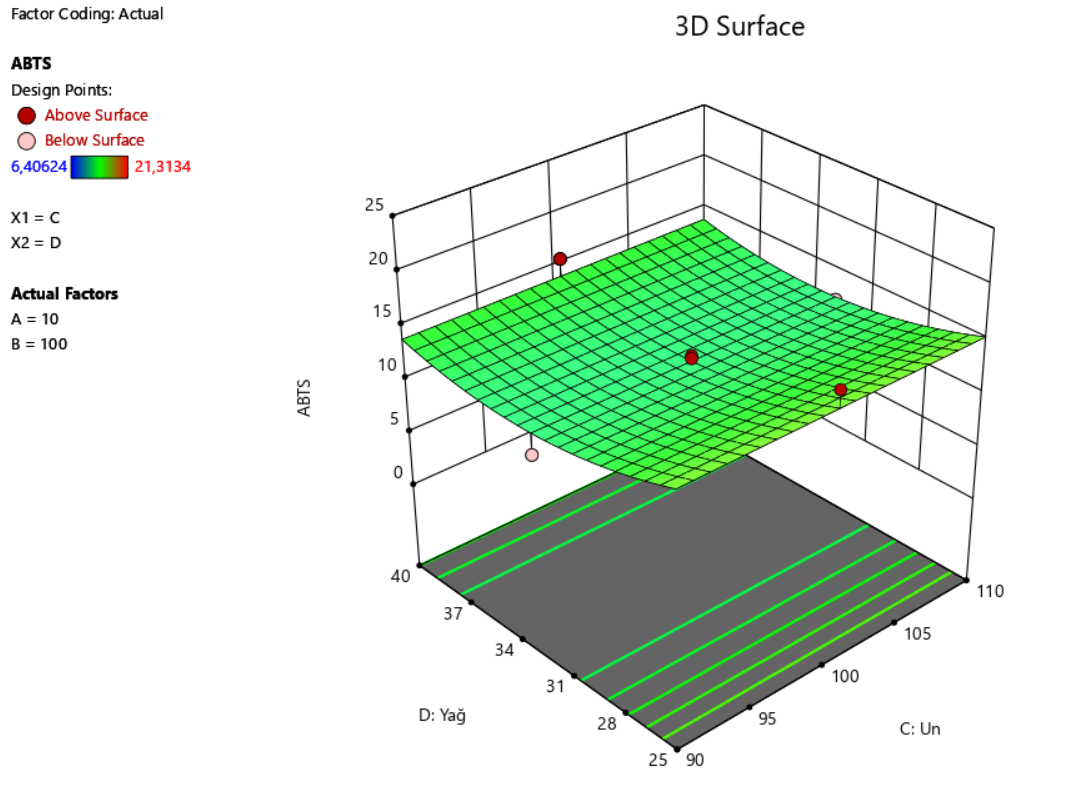


D: Oil

C: Flour

ABTS

ABTS

ABTS

ABTS

ABTS

A: Spirulina

A: Spirulina

B: Sugar

C: Flour

D: Oil

D: Oil

B: Sugar

C: Flour
